# Azithromycin sequential therapy plus inhaled terbutaline for *Mycoplasma Pneumoniae* pneumonia in children: a systematic review and meta-analysis

**DOI:** 10.1186/s12879-024-09564-x

**Published:** 2024-06-28

**Authors:** Yongcheng Sheng, Yi Liang, Chongyang Zhao, Deying Kang, Xueting Liu

**Affiliations:** 1 Chengdu Women’s and Children’s Central Hospital, School of Medicine, University of Electronic Science and Technology of China, Chengdu, Sichuan China; 2 West China School of Pharmacy, Sichuan University, Chengdu, Sichuan China; 3 Department of Evidence-based Medicine and Clinical Epidemiology, West China Hospital, Sichuan University, Chengdu, Sichuan China; 4 Center of Biostatistics, Design, Measurement and Evaluation (CBDME), Department of Clinical Research Management, West China Hospital, Sichuan University, Chengdu, Sichuan China; 5 West China Hospital, “Double First-class“ Construction office, Sichuan University, Chengdu, Sichuan China

**Keywords:** Azithromycin, Terbutaline, *Mycoplasma pneumoniae* pneumonia, Pulmonary ventilation function, Meta-analysis

## Abstract

**Background:**

An improper host immune response to *Mycoplasma pneumoniae* generates excessive inflammation, which leads to the impairment of pulmonary ventilation function (PVF). Azithromycin plus inhaled terbutaline has been used in the treatment of *Mycoplasma pneumoniae* pneumonia (MPP) in children with impaired pulmonary function, but previous randomized controlled trials (RCTs) showed inconsistent efficacy and safety. This study is aimed to firstly provide a systematic review of the combined therapy.

**Methods:**

This study was registered at the International Prospective Register of Systematic Reviews (PROSPERO CRD42023452139). A PRISMA-compliant systematic review and meta-analysis was performed. Six English and four Chinese databases were comprehensively searched up to June, 2023. RCTs of azithromycin sequential therapy plus inhaled terbutaline were selected. The revised Cochrane risk of bias tool for randomized trials (RoB2) was used to evaluate the methodological quality of all studies, and meta-analysis was performed using Stata 15.0 with planned subgroup and sensitivity analyses. Publication bias was evaluated by a funnel plot and the Harbord’ test. Certainty of evidence was assessed using the Grading of Recommendations, Assessment, Development and Evaluation recommendations.

**Results:**

A total of 1,938 pediatric patients from 20 RCTs were eventually included. The results of meta-analysis showed that combined therapy was able to significantly increase total effectiveness rate (RR = 1.20, 95%CI 1.15 to 1.25), forced expiratory volume in one second (SMD = 1.14, 95%CIs, 0.98 to 1.29), the ratio of forced expiratory volume in one second/forced vital capacity (SMD = 2.16, 95%CIs, 1.46 to 2.86), peak expiratory flow (SMD = 1.17, 95%CIs, 0.91 to 1.43). The combined therapy was associated with a 23% increased risk of adverse reactions compared to azithromycin therapy alone, but no significant differences were found. Harbord regression showed no publication bias (*P* = 0.148). The overall quality of the evidence ranged from moderate to very low.

**Conclusions:**

This first systematic review and meta-analysis suggested that azithromycin sequential therapy plus inhaled terbutaline was safe and beneficial for children with MPP. In addition, the combined therapy represented significant improvement of PVF. Due to lack of high-quality evidence, our results should be confirmed by adequately powered RCTs in the future.

**Supplementary Information:**

The online version contains supplementary material available at 10.1186/s12879-024-09564-x.

## Introduction

*Mycoplasma pneumoniae (M. pneumoniae)* is one of the most important pathogens for community-acquired pneumonia in hospitalized children [1]. As reported, *M pneumoniae* was detected in nearly 50% of the specimens of hospitalized children with acute respiratory infection in some cities of China [2]. It is estimated that approximately 5–12% of the hospitalized children with *Mycoplasma pneumoniae* pneumonia (MPP) were admitted to the intensive care unit [3]. An improper host immune response to *M. pneumoniae* generates excessive inflammation, which causes the impairment of pulmonary ventilation function (PVF) affecting both the large and small airways [4, 5].

According to the latest guidelines for MPP in China, macrolides represented by azithromycin have been the first-line antibiotics against *M. pneumoniae* [6]. To reduce complications such as pain and infection caused by intravenous injection, azithromycin sequential therapy (sequential switch from intravenous formulation to oral formulation) is applied as a new mode of antibiotic use in clinic. It is currently believed that MPP is a combination of a direct pathogen invasion and immune injury [7, 8].Thus, seeking for medication to reduce excessive immune reactions and improve PVF is urgently needed. Terbutaline has been widely used to improve lung resistance and reduce the incidence of respiratory distress, for example in asthma [9]. This ${{\beta }}_{2}$-agonist can relax bronchial smooth muscles, inhibit the release of endogenous spasmogenic substances and endogenous neurotransmitter-induced edema, and improve the clearance ability of bronchial mucosa cilia [10]. Terbutaline was well tolerated without irreversible adverse effects [9], and could decrease the incidence of acute respiratory failure in children with severe asthma exacerbations [11].

Currently, aerosols of terbutaline have been widely used in China [10], and there have been increasing number of randomized controlled trials (RCTs) on the efficacy and safety of azithromycin sequential therapy plus inhaled terbutaline for pediatric MPP, but they showed inconsistent results. This study is aimed to firstly provide a well-powered support on the combination therapy for MPP through a systematic review and meta-analysis.

## Materials and methods

### Protocol registration and reporting

The study protocol was registered at the International Prospective Register of Systematic Reviews (CRD42023452139). This systematic review was designed based on the Cochrane Handbook for Systematic Reviews of Interventions [12] and was reported in accordance with the Preferred Reporting Items for Systematic Reviews and Meta-analyses (PRISMA) statement [13] (see Appendix S11).

### Eligibility criteria

The strict **PICOS** framework is as follows:

#### (P) Participants

After laboratory tests and imaging tests, children (aged ≤ 16 years old) were diagnosed with MPP without any underlying serious disease or other acute infectious disease. Only the studies in which the clinical diagnostic criteria followed clinical guidelines or expert consensus were considered [14, 15]. Alternatively, the diagnosis of MPP was based on relevant clinical symptoms and chest imaging tests plus microbiologic tests by one of the following methods: culture, polymerase chain reaction (PCR) testing or serologic tests [6].

#### (I) Intervention

Azithromycin sequential therapy (sequential switch from intravenous formulation to oral formulation of azithromycin when the patient has an adequate clinical response and can efficiently absorb orally administered medication [16]) plus inhaled terbutaline was used for intervention group.

#### (C) Control

Azithromycin sequential therapy alone was used for control group. There was no restriction on drug dose in either group. When necessary, researchers should administer basic treatments such as reducing fever, relieving cough, expectoration, and so on.

#### (O) Outcomes

The primary outcomes were total effectiveness rate (TER) and the incidence of total adverse events (TAEs). The clinical responses were classified as cure (complete disappearance of all signs and symptoms of pneumonia with complete regression of infiltrate on control chest radiograph), improvement (defervescence with incomplete disappearance of other signs and symptoms of pneumonia with partial regression of infiltrate on control chest radiograph, and without need for additional antimicrobial therapy), or failure (lack of improvement or progression or recurrence of signs and symptoms of pneumonia after the treatment) [16]. Total effective rate = (number of cured patients + number of improved patients)/total number of patients× 100% [17]. TAEs mainly include nausea, vomiting, abdominal distension, abdominal pain, diarrhea, headache, rash and so on. The secondary outcomes included pulmonary function indexes, such as forced expiratory volume in one second (FEV1), the ratio of forced expiratory volume in one second/forced vital capacity (FEV1/FVC) and peak expiratory flow (PEF); Clinical symptom indexes: time to disappearance of fever, cough as well as lung rales, time to relief of asthma and return to normal of chest X-ray; Inflammation indicators: C-reactive protein (CRP), tumor necrosis factor-alpha (TNF-α), interleukin-2 (IL-2), interleukin-4 (IL-4), interleukin-6 (IL-6) and interleukin-8 (IL-8).

#### (S) Study design

RCTs published in English or Chinese. For different studies with the same research object, the latest research results were adopted.

### Exclusion criteria

The exclusion criteria are as follows:

1. Patients were treated with detailed interventions other than azithromycin and terbutaline in their studies, such as glucocorticoids, special nursing, and traditional Chinese medicine;
2. The treatment duration or efficacy assessment was unclear;
3. Patients who did not receive a clear standard of diagnosis or treatment;
4. Clinical studies that did not correctly describe randomized grouping methods. Their design was actually retrospective, observational study with controls.
5. There were no full text, incomplete articles, or duplicate publications.

### Search methods for identification of studies

Medline, Embase, Web of Science, Science Direct, Cochrane Library, Epistemonikos, CBM, CNKI, Wan-Fang and CQVIP databases were comprehensively searched up from database inception to June 2023. The search terms included: *Mycoplasma* pneumonia, *Mycoplasma* infection, MPP, primary atypical pneumonia, Mycoplasma ovipneumoniae infection, Mycoplasma dispar infection, azithromycin, and terbutaline (see Appendix S1 for full details of the search strategy).

All relevant studies were retrieved based on consensus, and the reference lists of the selected articles were further searched for additional relevant studies.

### Data collection and extraction

Two researchers (YCS and YL) independently completed the study screening, data extraction by using NoteExpress and Excel. Disagreements were resolved through discussion or with the assistance of DYK. Studies were initially screened based on the title and abstract, followed by full-text screening. The following basic data were extracted: (1) year of publication and first author’s name; (2) patient age; (3) course of disease; (4) sample size; (5) sex ratio; (6) therapy regime of the intervention and control groups; (7) treatment duration; and (8) details of outcomes.

### Assessment of risk of bias in included studies

The risk of bias of each included study was assessed using five dimensions of the revised Cochrane risk of bias tool for randomized trials (RoB2) [18]. Studies were graded as “low risk”, “some concerns”, or “high risk”, resulting in an overall bias assessment. YCS and YL independently rated all the articles. Conflicting ratings were discussed and resolved by consensus.

### Statistical analysis

The full meta-analysis was performed using Stata 15.0 and the results are represented as forest maps. Relative risk (RR) was used to calculate the effect for dichotomous variables, while standardized mean difference (SMD) was used for continuous variables. Each effect size provides a 95% confidence interval (CI). The heterogeneity among the studies was evaluated by Chi-square test (α = 0.1), and the differences in heterogeneity were evaluated by ${I}^{2}$. If there was no significantly statistical heterogeneity among the results, the fixed-effect model was used for statistical analysis. On the contrary, the main sources of relevant heterogeneity should be further analyzed. After excluding the influencing factors of clinical heterogeneity, the random-effect model was used for the statistical analysis. A rough choice between fixed- and random-effect meta-analysis was as follows: when${I}^{2}$ < 50%, a fixed-effect model was chosen to analyze the data. In contrast, when ${I}^{2}$≥ 50%, a random-effect model was used for statistical analysis. For all the test in these analyses, the statistical significance level was set in *P* < 0.05. If there was significant clinical heterogeneity, subgroup analysis or leave-one-out sensitivity analysis was used. A subgroup analysis with treatment durations was conducted. In addition, considering the quality of the original studies, we performed sensitivity analyses excluding trials at high risk of bias.

### Assessment of publication bias

To assess the risk of publication bias, we used funnel plots for visual inspection. Harbord regression was performed to assess publication bias, and if the hypothesis test *P* > 0.1, there was no significant publication bias.

### GRADE approach assessment

The Grading of Recommendations, Assessment, Development and Evaluation (GRADE) approach [19, 20] was used to assess the certainty of evidence in primary outcomes and pulmonary function indexes. The assessment criteria in the previous study were referred [21]. The GRADE approach categorizes the quality of evidence on four levels ranging from high to very low, with low levels of evidence indicating that future trials with high quality are likely to change the estimates.

## Results

### Description of studies

**Fig. 1:**
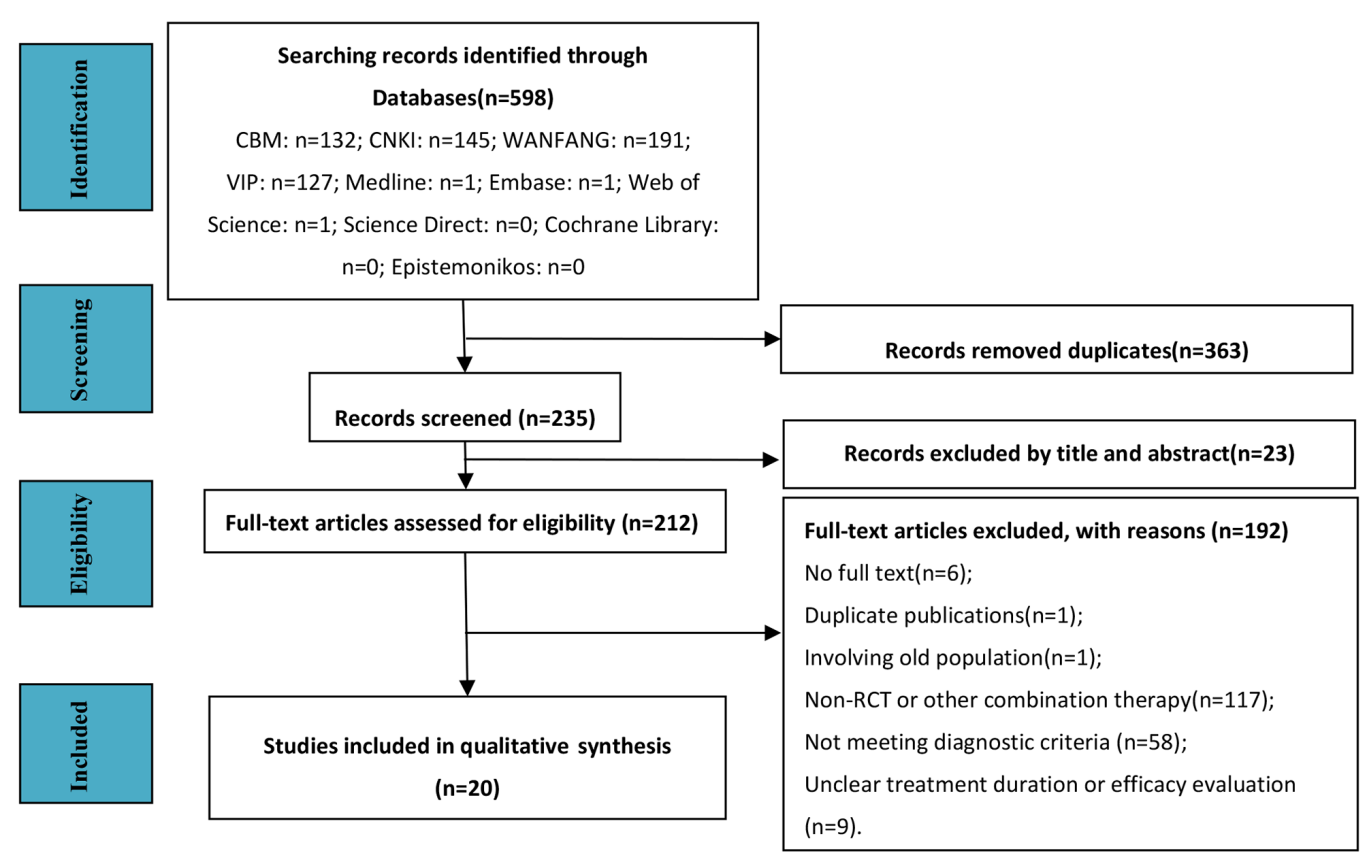
Flow diagram of studies considered for inclusion

The search process revealed 598 articles, and a total of 20 RCTs were ultimately included, as shown in Fig. 1 (see Appendix S2 for the full reference list). A total of 1,938 children (1,061 boys and 877 girls) participated, including 969 patients in the intervention group and 969 patients in the control group. They all came from China. All included children were patients with general MPP. The median age of the enrolled children was 5.32 years with a range of 0.33 ~ 13 years. The median course of disease was 4.00 days with a range of 1 ~ 28 days. Sixteen RCTs were conducted on one cycle of azithromycin sequential therapy (14 days) while 4 studies doubled the cycle of sequential therapy, that was 28 days. The common regimen of azithromycin sequential therapy was as follows: (1) azithromycin: 10 mg/(kg·d), qd (for 3–5 consecutive days), and ivgtt; (2) azithromycin therapy was stopped for 4 days; (3) azithromycin dry suspension: 10 mg/(kg·d), qd (for 3 days), and po; (4) azithromycin was stopped for 4 days. Terbutaline inhalation regimen was as follows: 2.5 mg (in 5 mL of 0.9% sodium chloride) at one time, and bid (the treatment time was the same as that of azithromycin). The basic characteristics of all included studies are shown in Table 1(see Appendix S3 for information on the diagnostic criteria and therapy regimens for each included RCT; see Appendix S4 for the characteristics of patient age and course among the 20 RCTs).

**Table 1:** Basic characteristics of the included RCTs.

No.	RCTs	Age/ $\overline X$ ± S, y		Gender, M/F		Course/ $\overline X$ ± S, d		Conventional therapy	C	Cycles of azithromycin sequential therapy*	I	Outcomes
		I	C	I	C	I	C					
1	Tong et al., 2021	5.88 ± 1.76	6.65 ± 1.82	22/18	19/21	-	-	#	A	1	A + B	①②③④⑥⑦⑧⑩⑫⑮
2	Wang, 2021a	4.46 ± 1.87	4.53 ± 1.82	22/20	20/22	5.46 ± 1.88	5.38 ± 1.92	#	A	1	A + B	①②⑥⑩⑫
3	Wang, 2021b	2.60 ± 1.30	2.80 ± 1.40	29/23	28/24	2.70 ± 0.60	2.80 ± 0.90	#	A	1	A + B	①⑥⑦⑧⑨⑪⑯
4	Wei et al., 2021	7.60 ± 2.70	7.10 ± 3.20	30/30	36/24	-	-	-	A	1	A + B	①②⑪⑫
5	Chen and Song, 2020	5.52 ± 2.23	5.58 ± 2.01	11/9	11/9	4.51 ± 0.65	5.58 ± 2.01	#	A	1	A + B	①②⑥⑦⑧⑨⑪⑫⑮
6	Chen, 2020	5.89 ± 3.23	5.82 ± 3.11	23/17	25/15	4.22 ± 2.23	4.23 ± 2.17	#	A	1	A + B	③④⑤⑬⑭
7	Cui et al., 2020	5.36 ± 1.96	5.84 ± 1.71	28/26	29/25	3.26 ± 1.40	3.93 ± 1.64	#	A	1	A + B	①②③④⑤⑥⑦⑧⑬⑭
8	Hao et al., 2020	5.51 ± 0.51	5.39 ± 0.43	18/16	20/14	4.05 ± 0.41	3.94 ± 0.35	#	A	1	A + B	①⑪
9	Li et al., 2019	4.30 ± 0.20	4.20 ± 0.30	57/43	52/48	4.30 ± 1.20	4.20 ± 1.10	#	A	1	A + B	①②⑥⑦⑧
10	Tong, 2019	6.98 ± 1.34	3.39 ± 1.94	24/28	27/25	3.07 ± 0.94	3.14 ± 0.72	-	A	1	A + B	①②③④⑤⑥⑦⑧⑨⑪⑫⑬
11	Cai, 2018	4.11 ± 1.45	4.21 ± 0.47	24/18	23/19	-	-	-	A	1	A + B	①②
12	Chen, 2018	3.42 ± 1.35	3.30 ± 1.41	28/20	26/22	3.32 ± 1.24	3.29 ± 1.31	-	A	1	A + B	①②③④⑤
13	Hou et al., 2018	3.35 ± 1.24	3.40 ± 1.19	24/21	26/19	3.98 ± 1.19	3.87 ± 1.31	-	A	1	A + B	①②③④⑤⑥⑦⑧⑨⑬⑭
14	Liang and Huang, 2018	6.91 ± 2.25	6.73 ± 2.17	25/19	26/18	18.08 ± 3.55	18.36 ± 3.43	-	A	1	A + B	②⑦⑧⑩⑪⑮⑯
15	Gong, 2017	7.90 ± 3.50	8.10 ± 3.60	30/21	28/23	-	-	-	A	1	A + B	①⑥⑦⑧⑫
16	Du et al., 2016	3.57 ± 1.13	4.16 ± 0.94	36/29	32/33	-	-	-	A	1	A + B	①②⑥⑦⑧⑨⑪⑮⑯
17	Liang, 2021	8.74 ± 2.35	8.54 ± 2.15	21/17	22/16	2.24 ± 0.02	2.24 ± 0.02	#	A	2	A + B	①②③④⑤⑥⑦⑧
18	Liu, 2021	4.20 ± 3.81	1.25 ± 3.76	23/14	21/16	-	-	-	A	2	A + B	①③④⑤⑥⑦⑧⑬⑭
19	Wang and Ren, 2019	3.29 ± 1.41	3.27 ± 1.58	38/24	37/25	4.21 ± 0.39	4.30 ± 0.27	-	A	2	A + B	①
20	Wang, 2018	6.01 ± 1.21	6.61 ± 1.03	19/24	23/20	-	-	-	A	2	A + B	①⑥⑦⑧⑪⑮⑯

RCTs, randomized controlled trials;$\overline X$ ± S, mean ± standard deviation; y, year; d, day; M, male; F, female; I, Intervention group; C, Control group; A, Azithromycin sequential therapy; B, Inhaled terbutaline; “-” denotes “not available”; “#” denotes “Conventional treatments such as correcting water electrolyte balance, resolving phlegm, reducing fever, and relieving cough”;“*” denotes “One cycle of azithromycin sequential therapy means 14 days”.①, Total effectiveness rate (TER); ②, Incidence of total adverse events (TAEs); ③, Forced expiratory volume in one second (FEV1); ④, Peak expiratory flow (PEF); ⑤, the ratio of Forced expiratory volume in one second/Forced vital capacity (FEV1 / FVC); ⑥, Time to disappearance of fever; ⑦, Time to disappearance of cough; ⑧, Time to disappearance of lung rales; ⑨, Time to relief of asthma; ⑩, Time for return to normal of chest X-ray; ⑪, Tumor necrosis factor alpha (TNF-α); ⑫, C-reactive protein (CRP); ⑬, Interleukin-2 (IL-2); ⑭, Interleukin-4 (IL-4); ⑮, Interleukin-6 (IL-6); ⑯, Interleukin-8 (IL-8)

### Risk of bias in included studies

**Fig. 2:**
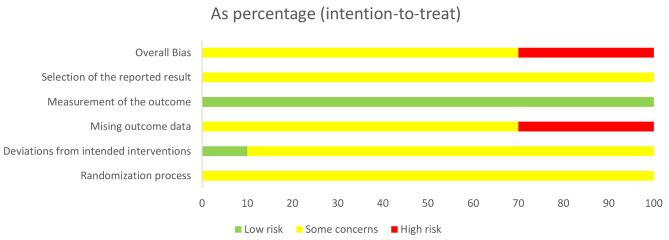
Overview of risk of bias assessment according to the revised Cochrane risk of bias tool for randomized trials (Rob2). Each colored area represents the percentage of studies in the respective bias assessments categories

Figure 2 showed an overview of the risk of bias assessment, and the quality of the included RCTs was overall moderate according to the intention-to-treat principle. A more detailed description of the risk of bias assessment for each study can be found in Appendix S5. Six studies (30%) were assessed to have an overall high risk of bias while fourteen (70%) were assessed to have “some concerns” regarding their overall bias. The most common reason for a high risk was missing outcome data (for incomplete drop-out information). However, there are several some potential risks of bias, such as inadequate blinding of assessors and selection of the reported result, due to the lack of pre-registration of RCTs.

### Results of primary outcomes

#### TER

**Fig. 3:**
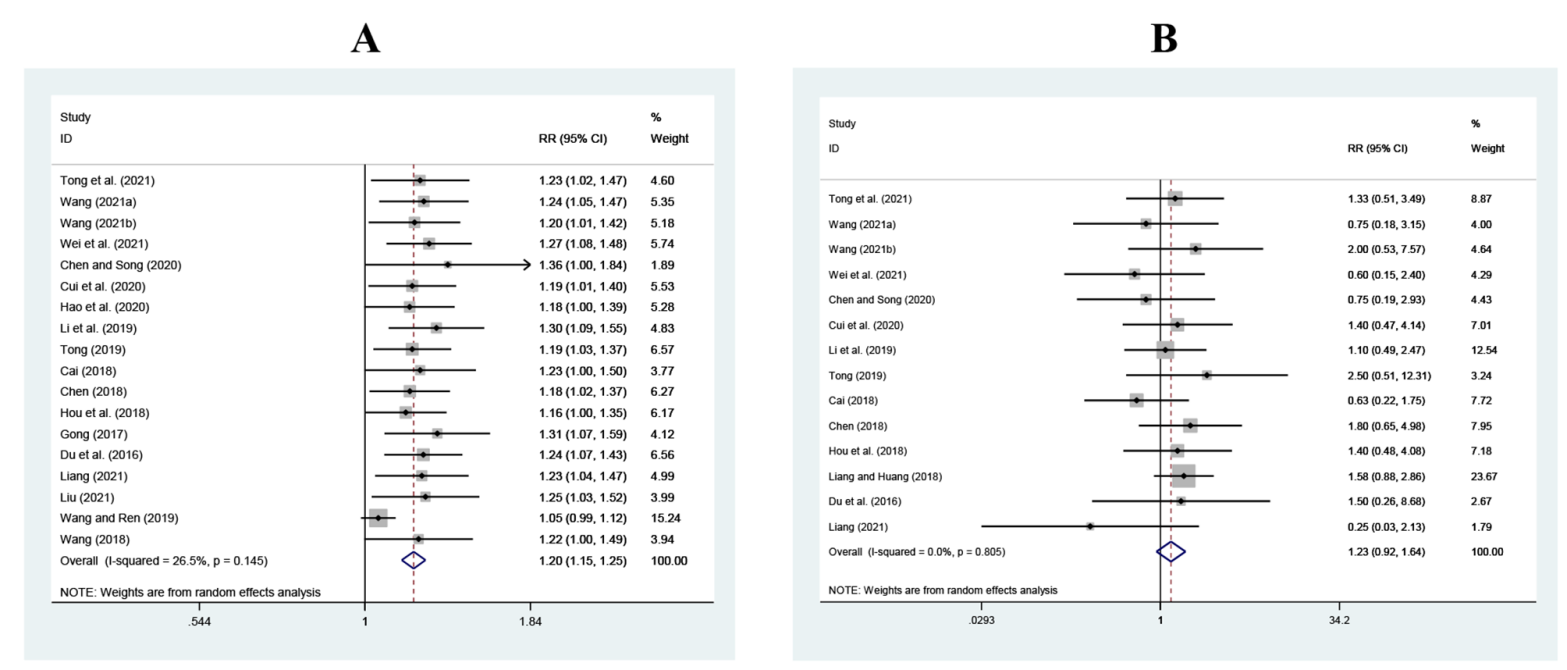
Results of meta-analysis for primary outcomes. **A**, Total effectiveness rate (TER); **B**, Incidence of total adverse events (TAEs)

A total of 18 RCTs reported TER, and the analysis included 885 patients in each group. The meta-analysis revealed no obvious heterogeneity (${I}^{2}$=26.5%, *P* = 0.145). Therefore, the fixed-effect model was selected for meta-analysis. The results showed that TER of the intervention group was significantly higher than that of the control group for the treatment of pediatric MPP (RR = 1.22, 95%CI 1.17 to 1.27, *Z* = 9.64, *P* < 0.001; Appendix S6.1 A). In fact, fixed-effect meta-analyses ignore heterogeneity. However, there are often many clinical characteristics that vary across studies. Thus, a random-effect model would be better for meta-analyses and we found that the result based on random-effect model changed slightly (RR = 1.20, 95%CI 1.15 to 1.25, Z = 8.06, *P* < 0.001; Fig. 3A).

#### Incidence of TAEs

The incidence of TAEs was evaluated according to the outcomes from 14 RCTs with 702 patients in each group. Regarding no obvious heterogeneity (${I}^{2}$=0.0%, *P* = 0.805), meta-analysis of fixed-effect model showed that the incidence of TAEs in the intervention group was higher than that of the control group in the treatment of MPP, but there was no significant difference between the two groups (RR = 1.20, 95%CI 0.91 to 1.59, *Z* = 1.27, *P* = 0.205; Appendix S6.1B). According to random-effect model, the confidence interval estimates were wider (RR = 1.23, 95%CI 0.92 to 1.64, Z = 1.41, *P* = 0.159; Fig. 3B). The main adverse events, such as nausea and vomiting, abdominal distension, abdominal pain, diarrhea, headache and rash, were common (1–10%) according to the criteria of the Council for International Organizations of Medical Sciences. These adverse reactions were mild and disappeared spontaneously after stopping the medication, and there was no significant difference in different adverse events between the two groups (see Appendix S10).

### Results of secondary outcomes

#### Outcomes of pulmonary function indexes

##### FEV1

**Fig. 4:**
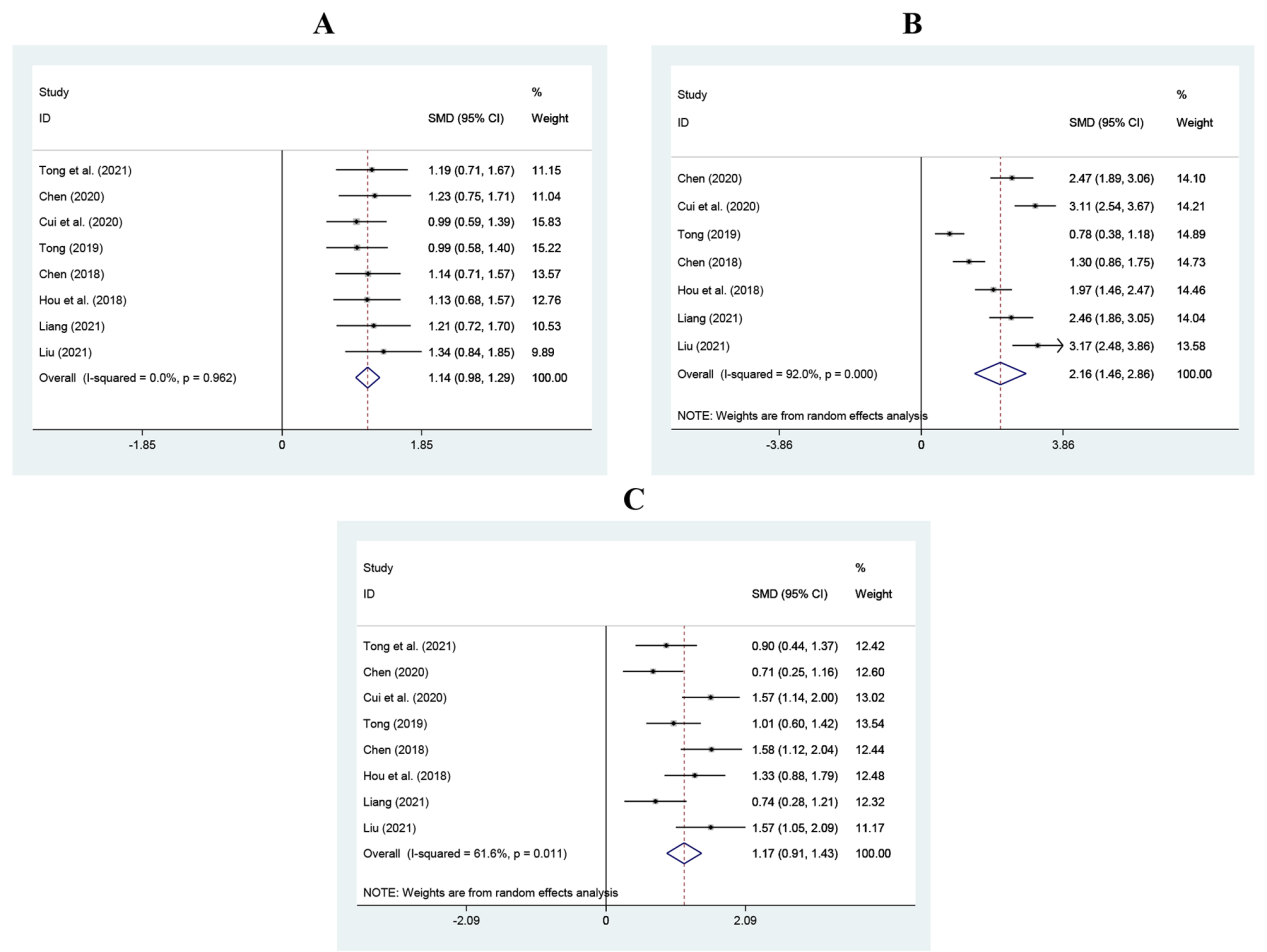
Results of meta-analysis for outcomes of pulmonary function indexes. **A**, Forced expiratory volume in one second (FEV1); **B**, the ratio of Forced expiratory volume in one second/Forced vital capacity (FEV1/FVC); **C**, Peak expiratory flow (PEF)

Eight RCTs with 354 patients in each group were included to investigate how azithromycin sequential therapy plus inhaled terbutaline affected FEV1. As no significant interstudy heterogeneity (${I}^{2}$=0%, *P* = 0.962) was observed, the fixed-effect model was used and the results showed that FEV1 of the intervention group was significantly higher than that of the control group (SMD = 1.14, 95%CI 0.98 to 1.29, *Z* = 9.64, *P* < 0.001; Fig. 4A).

##### Ratio of FEV1/FVC

Seven studies were included to investigate the difference in the ratio of FEV1/FVC between the two groups. The analysis showed that there were 314 patients in each group. There was obvious heterogeneity (${I}^{2}$=92.0%, *P*< 0.001), and meta-analysis results of random-effect model showed that the ratio of FEV1/FVC in the intervention group was significantly higher than that in the control group (SMD = 2.16, 95%CI 1.46 to 2.86, *Z* = 6.04, *P* < 0.001; Fig. 4B).

##### PEF

Eight RCTs with 354 patients in each group reported PEF. The random-effect model was selected for meta-analysis because of its obvious heterogeneity (${I}^{2}$=61.6%, *P* = 0.011) and PEF in the intervention group was significantly higher than that in the control group (SMD = 1.17, 95%CI 0.91 to 1.43, *Z* = 8.84, *P* < 0.001; Fig. 4C).

### Outcomes of clinical symptom indexes

A total of 13, 13, 13, 5 and 3 RCTs reported the time to disappearance of clinical symptoms, in term of fever, cough, lung rales, asthma relief, and return to a normal chest X-ray, respectively. The random-effect model was used due to relatively high heterogeneity, and the results demonstrated that the intervention group had significantly shorter time than the control group. The pooled results are shown in Table 2 and Appendix S6.

**Table 2:** Meta-analysis of clinical symptom indexes

Outcomes	Studies (*n*)	patients (*n*)	Pooled	Test of SMD	Heterogeneity	
			SMD (95% CI)	Z value, *P* value	${I}^{2}$(%)	*P* value
Time to disappearance of fever	13	1278	-1.25, 95%CI -1.62 to -0.88	Z = 6.60, *P* < 0.001	89.0	< 0.001
Time to disappearance of cough	13	1282	-2.24, 95%CI -2.89 to -1.60	Z = 6.81, *P* < 0.001	95.3	< 0.001
Time to disappearance of lung rales	13	1300	-1.55, 95%CI -1.93 to -1.18	Z = 8.08, *P* < 0.001	88.5	< 0.001
Time to relief of asthma	5	468	-1.50, 95%CI -2.04 to -0.96	Z = 5.42, *P* < 0.001	84.8	< 0.001
Time for return to normal of chest X-ray	3	252	-1.50, 95%CI -2.48 to -0.51	Z = 2.97, *P* = 0.003	91.7	< 0.001

### Outcomes of inflammatory indicators

There were respectively 6, 8, 5, 4, 5 and 4 RCTs reported levels of inflammatory indicators, namely, CRP, TNF-α, IL-2, IL-4, IL-6 and IL-8. Two indicators had no obvious heterogeneity but four outcomes did. The meta-analysis results of random-effect model showed an obviously increased level of IL-4 and lower levels of other inflammatory indicators in the intervention group than those in the control group. The pooled results are shown in Table 3 and Appendix S6.

**Table 3:** Meta-analysis of inflammatory indicators

Outcomes	Studies (*n*)	patients (*n*)	Pooled	Test of SMD	Heterogeneity	
			SMD (95% CI)	Z value, *P* value	${I}^{2}$(%)	*P* value
CRP	6	530	-1.48, 95%CI -2.07 to -0.90	Z = 4.96, *P* < 0.001	88.7	< 0.001
TNF-α	8	740	-1.27, 95%CI -1.65 to -0.89	*Z* = 6.55, *P* < 0.001	82.0	< 0.001
IL-2	5	456	-1.68, 95%CI -1.89 to -1.47	*Z* = 15.37, *P* < 0.001	0.0	0.927
IL-4	4	352	0.78, 95%CI 0.56 to 0.99	*Z* = 7.01, *P* < 0.001	0.0	0.525
IL-6	5	424	-2.00, 95%CI -3.14 to -0.86	*Z* = 3.44, *P* < 0.001	95.6	< 0.001
IL-8	4	408	-1.87, 95%CI -2.56 to -1.18	*Z* = 5.33, *P* < 0.001	88.2	< 0.001

C-reactive protein (CRP); Tumor necrosis factor alpha (TNF-α); Interleukin-2 (IL-2); Interleukin-4 (IL-4); Interleukin-6 (IL-6); Interleukin-8 (IL-8)

### Subgroup analysis of different treatment durations

We conducted subgroup analysis based on treatment duration using data from 14 RCTs with a duration of 14 days and 4 RCTs of with a duration of 28 days (see Appendix S7). Regarding the duration of 14 days, a random-effect meta-analysis using data from 14 RCTs yielded an RR of 1.22 for TER by the intervention group compared with the control group (95% CI 1.17 to 1.28, *Z* = 8.75, *P* < 0.001) with no heterogeneity (${I}^{2}$=0.0%, *P* for heterogeneity = 0.998). However, regarding the duration of 28 days, the pooled RR for TER in the intervention group compared with the control group was 1.17 (95%CI 1.01 to 1.25, *Z* = 2.16, *P* < 0.001) with substantial heterogeneity (${I}^{2}$=71.7%, *P* = 0.014). This subgroup analysis suggested that TER at14 days was higher than that at 28 days, and TER of both subgroups was significantly higher than that of the control group.

### Sensitivity analysis

We found that high heterogeneity appeared when conducting meta-analyses. Therefore, we further conducted leave-one-out sensitivity analysis to explore the potential source of heterogeneity, and there was no substantial modification of our estimates after the exclusion of individual studies one by one (see Appendix S8.1-2). The sensitivity analysis showed that the results were statistically robust. A further sensitivity analysis excluding all trials judged to be at high risk of bias confirmed these substantial results (see Appendix S8.3-6). Thus, we believe that the high heterogeneity may arise from the following factors: course of disease, treatment duration and the differences in measurements of the included trials.

### Publication bias

The funnel plot of TER was asymmetrical upon visual inspection while the funnel plot of the incidence of TAEs was symmetric. The results of the Harbord linear regression showed that there was a potential risk of publication bias in TER (*P* < 0.001) and no significant publication bias in the incidence of TAEs (*P* = 0.148; see Appendix S9).

### GRADE Certainty of evidence

The certainty of the evidence for the primary outcomes and pulmonary function indexes ranged from moderate to very low, as tabulated in the summary of findings table (Table 4). The reasons for downgrading the evidence included reporting bias, publication bias, inadequate precision, and heterogeneity.

**Table 4:** GRADE summary of findings

Outcome	Certainty assessment							Certainty
	№ of studies	Study design	Risk of bias	Inconsistency	Indirectness	Imprecision	Publication Bias	
TER	18	randomised trials	serious^a^	not serious	not serious	not serious	publication bias strongly suspected^b^	⨁⨁◯◯
								Low
Incidence of TAEs	14	randomised trials	serious^a^	not serious	not serious	not serious	none	⨁⨁⨁◯
								Moderate
FEV1	8	randomised trials	serious^a^	not serious	not serious	serious^c^	publication bias strongly suspected^d^	⨁◯◯◯
								Very Low
Ratio of FEV1/FVC	7	randomised trials	serious^a^	serious^e^	not serious	serious^c^	publication bias strongly suspected^d^	⨁◯◯◯
								Very Low
PEF	8	randomised trials	serious^a^	serious^e^	not serious	serious^c^	publication bias strongly suspected^d^	⨁◯◯◯
								Very Low

Explanations

a. Downgraded owing to risk of bias: Most information is from studies at low or unclear risk of bias, but potential limitations are likely to lower confidence in the estimate of effect

b. Downgraded owing to publication bias: The funnel plot was asymmetrical upon visual inspection

c. Downgraded owing to imprecision: Less than 400 participants included in meta-analysis

d. Downgraded owing to publication bias: Not possible to assess publication bias (less than 10 trials included)

e. Downgraded owing to inconsistency: *P* < 0.05(Chi-square test) and ${I}^{2}$value > 50%

## Discussion

In our study, the results showed that azithromycin sequential therapy plus inhaled terbutaline had higher efficacy (low-certainty evidence) and no more adverse reactions (moderate-certainty evidence) than azithromycin therapy alone.

This study showed that the combined therapy could increase the total efficacy by 20% compared to azithromycin therapy alone, suggesting that active treatment for MPP is highly important. Subgroup analysis of TER showed that different treatment durations could be potential causes of heterogeneity, but more RCTs of 28-day duration are needed to confirm these finding. In addition, no increase in efficacy was found after 28 days of therapy, which indicated that macrolide-resistant *M. pneumoniae* might develop after longer durations of azithromycin therapy and that patients cannot benefit from this therapy.

To avoid damage to pulmonary function, terbutaline was added to azithromycin sequential therapy to dilate the bronchus and promote pulmonary ventilation. Compared with the control treatment, the combined therapy improved FEV1 and the ratio of FEV1/FVC. Studies have shown that PEF can be used to screen impaired pulmonary function when spirometry is unavailable [22]. Our pooling results also showed that the intervention group had better PEF than the control group. The improvement in FVF could be confirmed by the time to the disappearance of cough, lung rales and asthma. The time of other clinical symptoms, such as fever and chest X-ray, also showed good improvement.

Host protein assays based on CRP are widely used in the diagnosis of community-acquired pneumonia, but CRP alone cannot be used to precisely separate bacterial from viral infection and mild from severe disease [23]. In the context of *M. pneumoniae* infection, it stimulates epithelial cells and macrophages to release a variety of cytokines, including pro-inflammatory cytokines (TNF-α, IL-2, IL-6, and IL-8) and anti-inflammatory cytokines (for example, IL-4) [7, 24]. The induction of pro-inflammatory cytokines is extensively involved in MPP development and can cause excessive immune inflammation, which includes local inflammation, airflow obstruction, air-way remodeling, emphysema and impaired lung function [24]. In our study, the levels of these pro-inflammatory cytokines in the intervention group were lower than those in the control group, suggesting that the combined therapy could downregulate the levels of pro-inflammatory factors and prevent the aggravation of MPP. Cytokines produced by ${\text{T}\text{h}}_{1}$ cells can be blocked by IL-4 produced by ${\text{T}\text{h}}_{2}$ cells and the ${\text{T}\text{h}}_{1}$/${\text{T}\text{h}}_{2}$ balance plays a significant role in anti-infectious immunity [25]. Therefore, the level of IL-4 in the intervention group was higher than that in the control group. The levels of pro-inflammatory cytokines decreased and anti-inflammatory cytokines increased in children with MPP, indicating that these cytokines are promising biomarkers for the diagnosis and treatment of this disease in clinical practice.

Compared with previous meta-analyses for MPP, there are a few points to be noted: Firstly, most of studies seemed to have unclear definitions of the diagnostic criteria for MPP [26–28], while the clinical symptoms of this disease are similar to those of viral and bacterial respiratory tract infections [5, 27], and Zhang et al [29]. even recruited patients regardless of the disease intensity. Secondly, the included interventions for MPP were not strict because extra medications such as acupuncture or special nursing were used in some studies [28, 30]. Thirdly, an observational study with controls, in which the patients were grouped by therapy regimens [27], could not be identified as an RCT, because it is highly likely that this was a retrospective analysis of clinical cases. In contrast, our study proposed a clear PICOS framework to decrease clinical and methodological heterogeneity.

To the best of our knowledge, this is the first systematic review and meta-analysis of azithromycin sequential therapy plus inhaled terbutaline for children with MPP. A comprehensive search was performed to obtain all relevant RCTs in our study and we paid more attention to pulmonary function indexes. Previous meta-analyses have reported overall response rate, total safety, improvement time of clinical symptoms and levels of inflammatory factors [31–33], while our study involved FEV1, the ratio of FEV1/FVC and PEF, which are the gold standards for the diagnosis of impaired pulmonary function [22]. To avoid misdiagnosis, pathogen identification was added as one of the inclusion criteria, which has never been highlighted before. We excluded some observational studies with controls to reduce methodological heterogeneity, which was different from previous meta-analyses [27]. This study was registered ahead of writing and performed in accordance with the PRISMA Checklist to reduce reporting bias as much as possible.

However, there were some limitations in our study. Firstly, the main limitation was the significant heterogeneity observed across the included studies. This heterogeneity remained in subgroup analyses of children stratified by treatment duration, which indicated that clinical characteristics such as age, onset time of MPP, and outcome measurements might be potential sources of bias. Although significant heterogeneity was detected, sensitivity analyses showed that the pooled results were statistically robust. Secondly, due to the overall moderate risk of bias and moderate to very low certainty of evidence, the results of this study should be interpreted with caution and high-quality RCTs are urgently needed. Thirdly, there was some risk in publication bias of TER and all included RCTs were conducted in China, whose underrepresentation could lead to inapplicable or non-generalizable results. More publications of relevant trials performed by researchers from different language backgrounds are needed to confirm the findings of the study. Fourthly, what element of any benefits attributed to terbutaline may in fact be a placebo in nature is difficult to estimate without blinded RCTs [34]. Fifthly, the inflammatory indicators including CRP, TNF-α, and IL-2 have potential prognostic value as diagnostic biomarkers for MPP, as well as make meta-analyses more valuable [35]. However, only a few studies have examined inflammatory indicators, which makes it difficult to analyze the immune response patterns of the host [36].

## Conclusions

This is the first systematic review and meta-analysis of azithromycin combined with terbutaline, which has shown good efficacy and safety. In addition, PVF and the levels of relevant indexes were significantly improved. However, there was no high-quality evidence and the results of this study should be interpreted with caution. Thus, we conditionally recommend azithromycin sequential therapy plus inhaled terbutaline for children with MPP. High-quality RCTs are needed to ensure safe medication practices for the combined therapy. In addition, the precise mechanism underlying effects of the combined therapy and inflammatory factors requires further in-depth study.

### Electronic supplementary material

Below is the link to the electronic supplementary material.

Supplementary Material 1

## Data Availability

All data generated or analyzed during the present study are included in this published article and Appendix.

## Abbreviations

MPP: *Mycoplasma pneumoniae* pneumonia
PVF: Pulmonary ventilation function
RCTs: Randomized controlled trials
PRISMA: The Preferred Reporting Items for Systematic Reviews and Meta-Analyses
PROSPERO: The International Prospective Register of Systematic Reviews
RoB2: The revised Cochrane risk of bias tool for randomized trials
TER: Total effectiveness rate
FEV1: Forced expiratory volume in one second
FEV1/FVC: Forced expiratory volume in one second/forced vital capacity
PEF: Peak expiratory flow
*M. pneumoniae*: *Mycoplasma pneumoniae*
PICOS: P–participants; I–intervention; C–control; O–outcomes; S–study design
TAEs: Total adverse events
CRP: C-reactive protein
TNF-α: Tumor necrosis factor-alpha
IL-2: Interleukin-2
IL-4: Interleukin-4
IL-6: Interleukin-6
IL-8: Interleukin-8
RR: Relative risk
SMD: Standardized mean difference
CI: Confidence interval
