# Supplementary material for: Azithromycin sequential therapy plus inhaled terbutaline for Mycoplasma Pneumoniae pneumonia in children: a systematic review and meta-analysis
Source: BMC Infect Dis. 2024 Jun 28;24:653. doi: 10.1186/s12879-024-09564-x (PMC11214698; doi:10.1186/s12879-024-09564-x)
Supplement: Supplementary file 1 — Supplementary Material 1 [file 12879_2024_9564_MOESM1_ESM.pdf]

Azithromycin Sequential Therapy plus Inhaled Terbutaline for *Mycoplasma Pneumoniae* Pneumonia in Children: A Systematic Review and Meta-analysis

Appendix to the manuscript

Contents of supplementary appendix

Appendix S1 Search strategy .... - 1 -
Appendix S2 References for included studies .... - 3 -
Appendix S3 Information of diagnostic criteria and therapy regime .... - 5 -
Appendix S4 Characteristics of patient age and course among 20 RCTs .... - 10 -
Appendix S5 Summary of risk of bias for included RCTs using RoB2 .... - 11 -
Appendix S6 Results of meta-analyses 12
Appendix S7 Subgroup analysis .... - 15 -
Appendix S8 Sensitivity analysis .... - 16 -
Appendix S9 Publication bias .... - 21 -
Appendix S10 Summary of adverse events .... - 22 -
Appendix S11 PRISMA 2020 Checklist .... - 24 -

Appendix S1 Search strategy

Search strategy for Ovid Medline

1. (Pneumonia, Mycoplasma).sh.
2. (Mycoplasma Pneumonia).ti.
3. (Mycoplasma Pneumonia).ab.
4. (Mycoplasma Pneumonia).kf.
5. (Mycoplasma Pneumonia).tc.
6. (Mycoplasma Pneumonia).kw.
7. (Mycoplasma infection).ti.
8. (Mycoplasma infection).ab.
9. (Mycoplasma infection).kf.
10. (Mycoplasma infection).tc.
11. (Mycoplasma infection).kw.
12. (MPP).ti.
13. (MPP).ab.
14. (MPP).kf.
15. (MPP).tc.
16. (MPP).kw.
17. (Primary Atypical Pneumonia).ti.
18. (Primary Atypical Pneumonia).ab.
19. (Primary Atypical Pneumonia).kf.
20. (Primary Atypical Pneumonia).tc.
21. (Primary Atypical Pneumonia).kw.
22. (Mycoplasma ovipneumoniae Infection).ti.
23. (Mycoplasma ovipneumoniae Infection).ab.
24. (Mycoplasma ovipneumoniae Infection).kf.
25. (Mycoplasma ovipneumoniae Infection).tc.
26. (Mycoplasma ovipneumoniae Infection).kw.
27. (Mycoplasma dispar Infection).ti.
28. (Mycoplasma dispar Infection).ab.
29. (Mycoplasma dispar Infection).kf.
30. (Mycoplasma dispar Infection).tc.
31. (Mycoplasma dispar Infection).kw.
32. or/1-31
33. (Azithromycin).sh.
34. (Azithromycin).ti.
35. (Azithromycin).ab.
36. (Azithromycin).kf.
37. (Azithromycin).tc.
38. (Azithromycin).kw.
39. or/33-28
40. (Terbutaline).sh.

41. (Terbutaline).ti.
42. (Terbutaline).ab.
43. (Terbutaline).kf.
44. (Terbutaline).tc.
45. (Terbutaline).kw.
46. or/40-45
47. 32 and 39 and 46

Search strategy for China Biology Medicine disc (CBM)

("支原体肺炎"[不加权:扩展] OR "原发非典型性肺炎"[中文标题:智能] OR "原发非典型性肺炎"[摘要:智能] OR "原发非典型性肺炎"[关键词:智能] OR "肺炎支原体"[中文标题:智能] OR "肺炎支原体"[摘要:智能] OR "肺炎支原体"[关键词:智能] OR "冷凝集阳性肺炎"[中文标题:智能] OR "冷凝集阳性肺炎"[摘要:智能] OR "冷凝集阳性肺炎"[关键词:智能] OR "支原体肺炎"[中文标题:智能] OR "支原体肺炎"[摘要:智能] OR "支原体肺炎"[关键词:智能] OR "支原体感染"[中文标题:智能] OR "支原体感染"[摘要:智能] OR "支原体感染"[关键词:智能]) AND ("阿奇霉素"[不加权:扩展] OR "阿奇霉素"[中文标题:智能] OR "阿奇霉素"[摘要:智能] OR "阿奇霉素"[关键词:智能]) AND ("特布他林"[不加权:扩展] OR "特布他林"[中文标题:智能] OR "特布他林"[摘要:智能] OR "特布他林"[关键词:智能] OR "特步他林"[中文标题:智能] OR "特步他林"[摘要:智能] OR "特步他林"[关键词:智能])

Appendix S2 References for included studies

1. Cai RR. Effect and the incidence of adverse reactions of azithromycin combined with terbutaline spray inhalation on mycoplasma pneumonia in children. *Clinical Research and Practice* 2018; 3: 87-88.
2. Chen JL. Effect of terbutaline-assisted azithromycin sequential therapy in children with mycoplasma pneumonia and the influence on airway function. *Clinical Research and Practice* 2018; 3: 109-110.
3. Chen Y and Song LM. Evaluation of therapeutic value of terbutaline combined with azithromycin in the treatment of mycoplasma pneumonia in children. *Chinese Community Doctors* 2020; 36: 18-19.
4. Chen Y. Effects of terbutaline on pulmonary function and inflammatory factors in children with mycoplasma pneumonia. *Chin Pediatr Integr Tradit West Med* 2020; 12: 72-74.
5. Cui XH, Zhao JY and Ma L, et al. Clinical Observation of Azithromycin Combined with Terbutaline Sequential Therapy in the Treatment of Mycoplasma Pneumoniae Pneumonia in Children. *Journal. Of Ningxia Medical. University* 2020; 42: 71-75.
6. Du XN, Liang LX and Yan HF, et al. Clinical Observation of Azithromycin Sequential Therapy Combined with Terbutaline in the Treatment of Mycoplasma Pneumonia. *China Pharmacy* 2016; 27: 740-742.
7. Gong YM, Li J and Mei P. Efficacy analysis of azithromycin combined with terbutaline nebulized inhalation in the treatment of pediatric mycoplasma pneumonia. *Journal of Clinical Medicine in Practice* 2017; 21: 159-161.
8. Hao YQ, Li YL and Wei F. Effects of terbutaline atomization inhalation combined with azithromycin sequential therapy on TNF-α level and lung function in children with Mycoplasma pneumonia. *Clinical Research and Practice* 2020; 5: 61-63.
9. Hou X, Jin R and Xie Y. Clinical study of terbutaline in treatment of children with mycoplasma pneumonia. *Journal of Clinical Pulmonary Medicine* 2018; 23: 1439-1442.
10. Li HY, Huang WW and Guan RM, et al. Effect of Azithromycin Combined with Terbutaline Sequential Treatment for Mycoplasma Pneumonia in Children. *Henan Medical Research* 2019; 28: 4483-4484.
11. Liang LH and Huang H. Efficacy of Azithromycin and Terbutaline in the Treatment of Children Mycoplasma Pneumonia. *Drugs and Clinic* 2018; 15: 21-23.
12. Liang LY. Effect of Azithromycin and Terbutaline in Sequential Treatment of Mycoplasma Pneumonia in Children and Its Influence on Pulmonary Function. *China Health Standard Management* 2021; 12: 90-93.
13. Liu F. The Effect of Azithromycin Combined with Terbutaline Nebulized Inhalation in the Treatment of Mycoplasma Pneumonia in Children. *System Medicine* 2021; 6: 92-94.
14. Tong JM. Clinical Effect of TerbutaIine in the Treatment of Children with Mycoplasmal Pneumonia and Its Effect on Serum Inflammatory Factors. *Chinese Medical Innovations* 2019; 16: 1-5.
15. Tong RX, Chen Q and Chang ZT, et al. Effect of Azithromycin Sequential Therapy Combined with Terbutaline Sulfate on Pulmonary Function and Serum IL-6, CRP and PCT Levels in Children

with Mycoplasma Pneumonia. *Progress in Modern Biomedicine* 2021; 21: 3160-3163.
16. Wang C and Ren DX. Effect of Terbutaline Nebulization Inhalation Combined with Azithromycin Sequential Treatment on Respiratory, Immune Function, and Clinical Efficacy in Children with Mycoplasma Pneumonia. *Chin J of Clinical Rational Drug Use* 2019; 12: 89-90.
17. Wang J. Effect of Azithromycin combined with terbutaline in the treatment of mycoplasma pneumonia and its influence on CRP and WBC. *Capital Food Medicine* 2021a; 28: 53-54.
18. Wang M. Effect of azithromycin sequential therapy and terbutaline on children with mycoplasma pneumonia and its impact on cytokines levels. *Clinical Research and Practice* 2018; 3: 81-82.
19. Wang ZC, Liu JX and Chen WQ. Clinical effect of terbutaline on Mycoplasma pneumonia in children and its impact on CXCL8 and P2X7 receptor levels. *J of Wannan Medical College* 2021b; 40: 136-139.
20. Wei LJ, Zhou JY and Shen JN. The efficacy of azithromycin combined with terbutaline in the treatment of mycoplasma pneumonia in children and its impact on serum levels of inflammatory cytokines and T lymphocyte subsets. *Maternal and Child Health Care of China* 2021; 36: 3988-3990.

Appendix S3 Information of diagnostic criteria and therapy regime

Table S3.1 Information of diagnostic criteria

No.	RCTs	Diagnostic criteria
1	Tong et al., 2021	(1) Referring to the diagnostic criteria of MPP in *Zhu Futang Practical Pediatrics*; (2) MP antibodies shows positive; (3) The clinical symptoms include rough respiratory sounds during auscultation of both lungs, elevated body temperature, and irritating dry cough. Imaging examination shows the lesions mostly with blurry edges and low-density cloud like infiltrating shadows.
2	Wang, 2021a	In accordance with the diagnostic criteria for pediatric mycoplasma pneumonia in *Pediatrics (9th edition)*, all children underwent imaging examinations, pathogen identification, and other examinations.
3	Wang, 2021b	In accordance with the diagnostic criteria for MPP in the "*Guidelines for Community Pneumonia Management in Children (2013 Revision)*" and the disease has been identified using chest X-ray and laboratory polymerase chain reaction (PCR).
4	Wei et al., 2021	In accordance with the diagnostic criteria of mycoplasma pneumonia in children in *Zhu Futang Practical Pediatrics*.
5	Chen and Song, 2020	All children's serum specific antibody IgM test results were positive.
6	Chen, 2020	Referring to the diagnostic criteria for Mycoplasma pneumonia in the "*Expert Consensus on the Diagnosis and Treatment of Mycoplasma Pneumonia in Children (2015)*".
7	Cui et al., 2020	All patients were diagnosed based on clinical symptoms, chest X-ray findings, laboratory tests, and met the diagnostic criteria of the "*Expert Consensus on the Diagnosis and Treatment of Mycoplasma Pneumonia in Children (2015 Edition)*".
8	Hao et al., 2020	In accordance with the diagnostic criteria of MP in *Zhu Futang Practical Pediatrics*. The children all had fever, persistent severe cough, sore throat and other symptoms and were confirmed by imaging examination.
9	Li et al., 2019	According to the clinical diagnostic criteria of Mycoplasma pneumoniae pneumonia in *Zhu Futang Practical Pediatrics*, it was confirmed as Mycoplasma pneumoniae pneumonia by laboratory examination and imaging examination; The children present with typical symptoms such as fever and cough.
10	Tong, 2019	The symptoms and auxiliary examination results comply with the clinical diagnostic criteria for pediatric mycoplasma pneumonia in the "*Expert Consensus on the Diagnosis and Treatment of Mycoplasma Pneumonia in Children (2015 Edition)*".
11	Cai, 2018	① Serum specific antibody IgM shows positive; ② Fever and discomfort lasting for more than 5 days, accompanied by coughing; ③ Chest X-ray shows thickening of bilateral lung markings with patchy shadows; ④ Some children were accompanied by Pleural effusion, and the auscultation of the lungs was accompanied by obvious dry and wet rales.

12	Chen, 2018	In accordance with the clinical diagnostic criteria for pediatric Mycoplasma pneumoniae pneumonia in the "*Expert Consensus on the Diagnosis and Treatment of Mycoplasma Pneumonia in Children (2015 Edition)*".
13	Hou et al., 2018	① In accordance with the diagnostic criteria for Mycoplasma pneumoniae pneumonia in the "*Expert Consensus on the Diagnosis and Treatment of Mycoplasma Pneumonia in Children (2015 Edition)*"; ② All are initial cases of mycoplasma pneumonia.
14	Liang and Huang, 2018	① All patients were diagnosed through X-ray and CT examinations; ② IgM test for Mycoplasma pneumoniae antibody is positive; ③ All are accompanied by clinical manifestations such as cough, fever, and sore throat.
15	Gong, 2017	In accordance with the diagnostic criteria of mycoplasma pneumonia in children in *Zhu Futang Practical Pediatrics*.
16	Du et al., 2016	All children were tested positive for serum specific antibody (IgM). The children developed fever and discomfort lasting for more than 5 days, accompanied by cough. Chest X-ray examination showed that bilateral lung markings were thickened, accompanied by small patches or patchy shadows, or even large shadows. A small number of pediatric patients developed atelectasis, Pleural effusion, etc. The lungs could be auscultated and the breath sounds were thickened, and some children had dry and wet rales.
17	Liang, 2021	In accordance with the diagnostic criteria for Mycoplasma pneumonia in *Practical Pediatrics*; The serum specific antibody IgM test is positive
18	Liu, 2021	All patients were diagnosed as MPP through IgM test, chest X-ray test and lung auscultation; Cough, fever, and other symptoms persist for more than 5 days.
19	Wang and Ren, 2019	The symptoms of fever, cough, shortness of breath and chest pain persisted for more than 5 days. The serum specific antibody IgM was diagnosed as positive, and the chest X-ray scan showed thickening of bilateral lung markings with patchy and small spot like shadows. Auscultation could also detect coarse aspirated sounds and dry and wet rales.
20	Wang, 2018	Serum specific antibody IgM was positive; Fever and discomfort lasting for more than 5 days, accompanied by coughing; Chest X-ray shows thickening of bilateral lung markings with patchy shadows; Some children were accompanied by Pleural effusion, and the auscultation of the lungs was accompanied by obvious dry and wet rales.

Table S3.2 Information of therapy regimes

No.	RCTs	Control group	Cycles of azithromycin sequential therapy	Intervention group
1	Tong et al., 2021	A therapy: ①Azithromycin: 10mg/(kg · d), qd (for 3-5 consecutive days), ivgtt; ② then stopped for 4 days; ③Azithromycin dry suspension: 10mg/(kg · d), qd (for 3 days), po.	1	A therapy+<br>B therapy: Terbutaline sulfate inhalation: 2.5mg at one time, bid, continuous use for 7-10 days.
2	Wang, 2021a	A therapy: ①Azithromycin: 10mg/(kg · d), qd (for 3 consecutive days), ivgtt; ② then stopped for 4 days; ③Azithromycin dry suspension: 10mg/(kg · d), qd (for 3 days), po (one hour before meals or two hours after meals); ④ the drug was stopped for 4 days.	1	A therapy+<br>B therapy: Terbutaline sulfate inhalation: 2.5mg (weight < 20kg) or 5mg (weight ≥20kg) at one time, bid. The treatment duration is the same as that of Azithromycin.
3	Wang, 2021b	A therapy: ①Azithromycin: 10mg/(kg · d) (in normal saline to maintain the concentration of 1~2g/L for intravenous drip), qd (for 3-5 consecutive days), ivgtt; ② then stopped for 4 days; ③ Azithromycin dispersible tablet: 10mg/(kg · d), qd (for 3 days), po. If there is an aggravation of the disease, appropriate intravenous drip of Lactobionic acid Erythromycin, 30 mg/kg.	1	A therapy+<br>B therapy: Terbutaline sulfate inhalation: 2.5mg (in 5mL of 0.9% sodium chloride) at one time (flow rate at 6-8L/min, 10-15min each time), bid.
4	Wei et al., 2021	A therapy: ①Azithromycin: 10mg/(kg · d) (in 5% glucose injection), qd (for 3 consecutive days), ivgtt; ② then stopped for 4 days; ③If the patient's condition is stable, it will be changed to oral Azithromycin dry suspension: 10mg/(kg · d), qd (for 3 days), po; ④ the drug was stopped for 4 days.	1	A therapy+<br>B therapy: Terbutaline sulfate inhalation: 2.5mg (weight < 20kg) or 5mg (weight ≥20kg) at one time, bid. The treatment duration is the same as that of Azithromycin.
5	Chen and Song, 2020	A therapy: ①Azithromycin: 10mg/(kg · d), qd (for 5 days), ivgtt; ② then stopped for 4 days; ③Azithromycin granules: 10mg/(kg · d), qd (for 3 days), po.	1	A therapy+<br>B therapy: Terbutaline inhalation: 2.5mg (weight < 20kg) at one time, qd; or 5mg (weight ≥20kg) at one time, tid. Continuous use for 7 days.

6	Chen, 2020	A therapy: ①Azithromycin: 10mg/(kg · d) (in 250mL of 0.9% sodium chloride), qd (for 5 consecutive days), ivgtt; ② then stopped for 4 days; ③oral Azithromycin: 10mg/(kg · d), qd (3 days for mild cases, 5 to 7 days for severe cases), po; ④ the second course of treatment could be repeated after 4 days.	1	A therapy+<br>B therapy: Terbutaline inhalation: 2.5mg (in 0.9% sodium chloride) at one time (10 min each time), bid. Continuous use for 14 days.
7	Cui et al., 2020	A therapy: ①Azithromycin: 10mg/(kg · d), qd (for 5 consecutive days), ivgtt; ② then stopped for 4 days; ③Azithromycin granules: 10mg/(kg · d), qd (for 5 days), po.	1	A therapy+<br>B therapy: Terbutaline sulfate inhalation: 2.5mg (in 5mL of 0.9% sodium chloride) at one time, bid. The treatment duration is the same as that of Azithromycin.
8	Hao et al., 2020	A therapy: ①Azithromycin: 10mg/(kg · d) (in 250mL of 5% glucose solution), qd (for 3-5 consecutive days), ivgtt; ② Azithromycin dispersible tablet: 10mg/(kg · d), qd (for 3 days), po.	1	A therapy+<br>B therapy: Terbutaline sulfate inhalation: 2.5mg (in 3mL of 0.9% sodium chloride) at one time (10-15min each time), bid.
9	Li et al., 2019	A therapy: ①Azithromycin: 10mg/(kg · d), qd (for 7 days), ivgtt; ② then stopped for 3 days; ③Azithromycin: 10mg/(kg · d), qd (for 4 days), po.	1	A therapy+<br>B therapy: Terbutaline inhalation: 2.5mg at one time, bid. Continuous use for 7 days.
10	Tong, 2019	A therapy: ①Azithromycin: 10mg/(kg · d) (in 5% glucose solution, concentration: 1 mg/mL), qd (for 5 days), ivgtt; ② then stopped for 4 days; ③Azithromycin dry suspension (<10 years) or Azithromycin tablets (>10 years): 10mg/(kg · d), qd (for 5 days), po.	1	A therapy+<br>B therapy: Terbutaline sulfate inhalation: 2.5mg (in 5mL of 0.9% sodium chloride). The treatment duration is the same as that of Azithromycin.
11	Cai, 2018	A therapy: ①Azithromycin: 10mg/(kg · d), qd (for 3-5 consecutive days), ivgtt; ② then stopped for 4 days; ③Azithromycin dry suspension: 10mg/(kg · d), qd (for 3 days), po.	1	A therapy+<br>B therapy: Terbutaline inhalation: 2.5mg (in 5mL of 0.9% sodium chloride) at one time (flow rate at 6-8L/min, 10-15min each time), bid.
12	Chen, 2018	A therapy: ①Azithromycin: 10mg/(kg · d) (in 250mL of 0.9% sodium chloride), qd (for 5 days), ivgtt; ② then stopped for 4 days; ③Azithromycin granules: 10mg/(kg · d), qd (for 5 days), po.	1	A therapy+<br>B therapy: Terbutaline inhalation: 2.5mg (in 4mL of 0.9% sodium chloride) at one time, bid. The treatment duration is the same as that of Azithromycin.
13	Hou et al., 2018	A therapy: ①Azithromycin: 10mg/(kg · d), qd (for 5 days), ivgtt; ② then stopped for 4 days; ③Azithromycin granules: 10mg/(kg · d), qd (for 5 days), po.	1	A therapy+<br>B therapy: Terbutaline inhalation: 2.5mg (in 4mL of 0.9% sodium chloride) at one time (10 min each time), bid. The treatment duration is the same as that of Azithromycin.

14	Liang and Huang, 2018	A therapy: ①Azithromycin: 6-10 mg/(kg · d), qd (for 3-5 consecutive days), ivgtt; ② then stopped for 4 days; ③ Azithromycin: 10mg/(kg · d), qd (for 7 days), po.	1	A therapy+<br>B therapy: Terbutaline inhalation: bid. Continuous use for 14 days.
15	Gong, 2017	A therapy: ①Azithromycin: 10 mg/(kg · d), qd (for 3-5 consecutive days), ivgtt; ② then stopped for 4 days; ③ Azithromycin dry suspension: 10mg/(kg · d), qd (for 3 days), po; ④ the drug was stopped for 4 days.	1	A therapy+<br>B therapy: Terbutaline sulfate inhalation: 2.5mg (weight < 20kg) or 5mg (weight ≥20kg) at one time, bid.
16	Du et al., 2016	A therapy: ①Azithromycin: 10 mg/(kg · d), qd (for 3-5 consecutive days), ivgtt; ② then stopped for 4 days; ③ Azithromycin dispersible tablets: 10mg/(kg · d), qd (for 3 days), po; ④ the drug was stopped for 4 days.	1	A therapy+<br>B therapy: Terbutaline sulfate inhalation: 2.5mg (in 5mL of 0.9% sodium chloride) at one time (flow rate at 6-8L/min, 10-15min each time), bid.
17	Liang, 2021	A therapy: ①Azithromycin: 10mg/(kg · d) (in 250mL of 0.9% sodium chloride), qd (for 5 days), ivgtt; ② then stopped for 4 days; ③Azithromycin: 10mg/(kg · d), qd (3 days for mild cases, 5 days for severe cases), po; ④ the drug was stopped for 4 days.	2	A therapy+<br>B therapy: Terbutaline sulfate inhalation: 5mg (in 50 mL of 0.9% sodium chloride) at one time (flow rate at 6-8L/min, 10-15min each time).
18	Liu, 2021	A therapy: ①Azithromycin: 10 mg/(kg · d), qd (for 4 days), ivgtt; ② then stopped for 4 days; ③ Azithromycin dispersible tablets: 10mg/(kg · d), qd (for 3 days), po; ④ the drug was stopped for 4 days.	2	A therapy+<br>B therapy: Terbutaline inhalation: 2.5mg (in 5mL of 0.9% sodium chloride) at one time (flow rate at 7 L/min, 15min each time), bid.
19	Wang and Ren, 2019	A therapy: ①Azithromycin: 10 mg/(kg · d), qd (for 3-5 consecutive days), ivgtt; ② then stopped for 4 days; ③ Azithromycin: 10mg/(kg · d), qd (for 3 days), po; ④ the drug was stopped for 4 days.	2	A therapy+<br>B therapy: Terbutaline inhalation: 2.5mg (in 5 mL of 0.9% sodium chloride) at one time (flow rate at 6-8L/min, 15min each time), bid.
20	Wang, 2018	A therapy: ①Azithromycin: 10 mg/(kg · d), qd (for 3-5 consecutive days), ivgtt; ② then stopped for 4 days; ③ Azithromycin dispersible tablets: 10mg/(kg · d), qd (for 3 days), po; ④ the drug was stopped for 4 days.	2	A therapy+<br>B therapy: Terbutaline inhalation: 1.5mg (in 5 mL of 0.9% sodium chloride) at one time (flow rate at 6-8L/min, 10-15min each time), bid. Continuous use for 28 days.

A therapy, Azithromycin sequential therapy; **B therapy**, Inhaled terbutaline.

Appendix S4 Characteristics of patient age and course among 20 RCTs

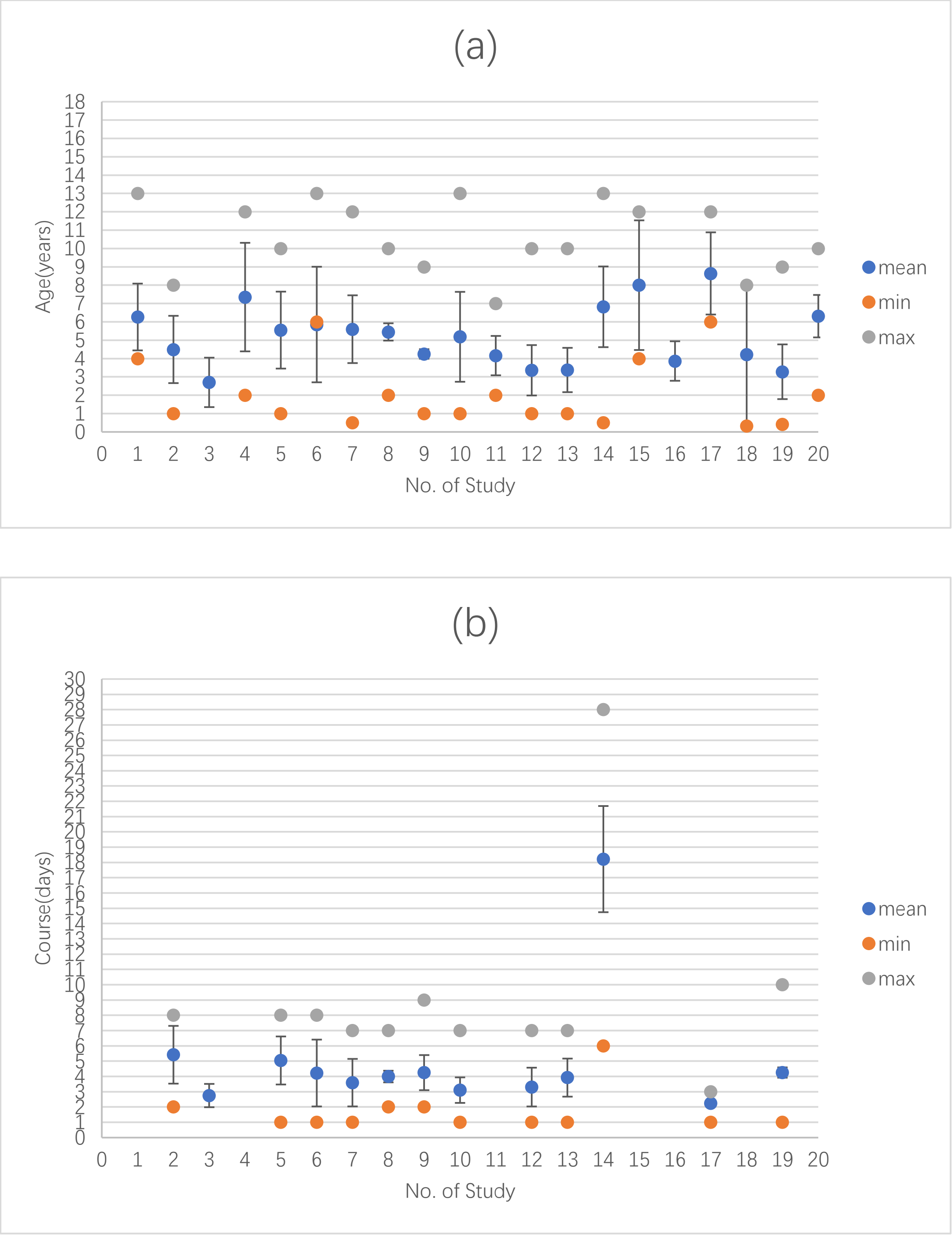

Figure S4. Characteristics of patient age and course among 20 RCTs.

Appendix S5 Summary of risk of bias for included RCTs using RoB2

Unique ID	Weight	D1	D2	D3	D4	D5	Overall
Tong et al., 2021	1	!	!	-	+	!	-
Wang, 2021a	1	!	!	-	+	!	-
Wang, 2021b	1	!	!	!	+	!	!
Wei et al., 2021	1	!	!	!	+	!	!
Chen and Song, 2020	1	!	!	!	+	!	!
Chen, 2020	1	!	+	-	+	!	-
Cui et al., 2020	1	!	!	-	+	!	-
Hao et al., 2020	1	!	!	!	+	!	!
Li et al., 2019	1	!	!	!	+	!	!
Tong, 2019	1	!	!	-	+	!	-
Cai, 2018	1	!	!	!	+	!	!
Chen, 2018	1	!	!	!	+	!	!
Hou et al., 2018	1	!	!	!	+	!	!
Liang and Huang, 2018	1	!	+	!	+	!	!
Gong, 2017	1	!	!	-	+	!	-
Du et al., 2016	1	!	!	!	+	!	!
Liang, 2021	1	!	!	!	+	!	!
Liu, 2021	1	!	!	!	+	!	!
Wang and Ren, 2019	1	!	!	!	+	!	!
Wang, 2018	1	!	!	!	+	!	!

+ Low risk
! Some concerns
- High risk

D1 Randomisation process
D2 Deviations from the intended interventions
D3 Missing outcome data
D4 Measurement of the outcome
D5 Selection of the reported result

Figure S5. Summary of risk of bias for included RCTs using RoB2.

Appendix S6 Results of meta-analyses

A   B

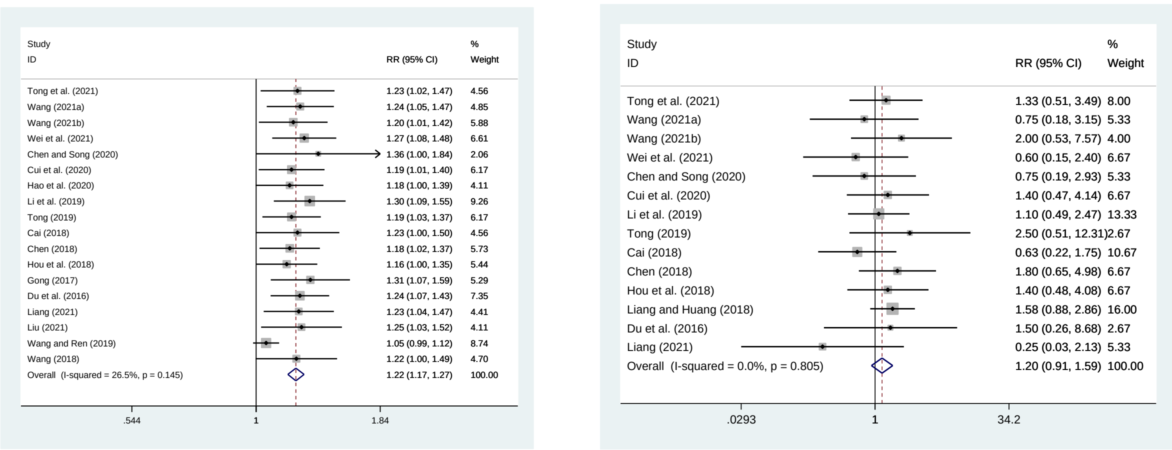

Figure S6.1. Results of meta-analysis for primary outcomes. A, Total effectiveness rate (TER); B, Incidence of total adverse events (TAEs).

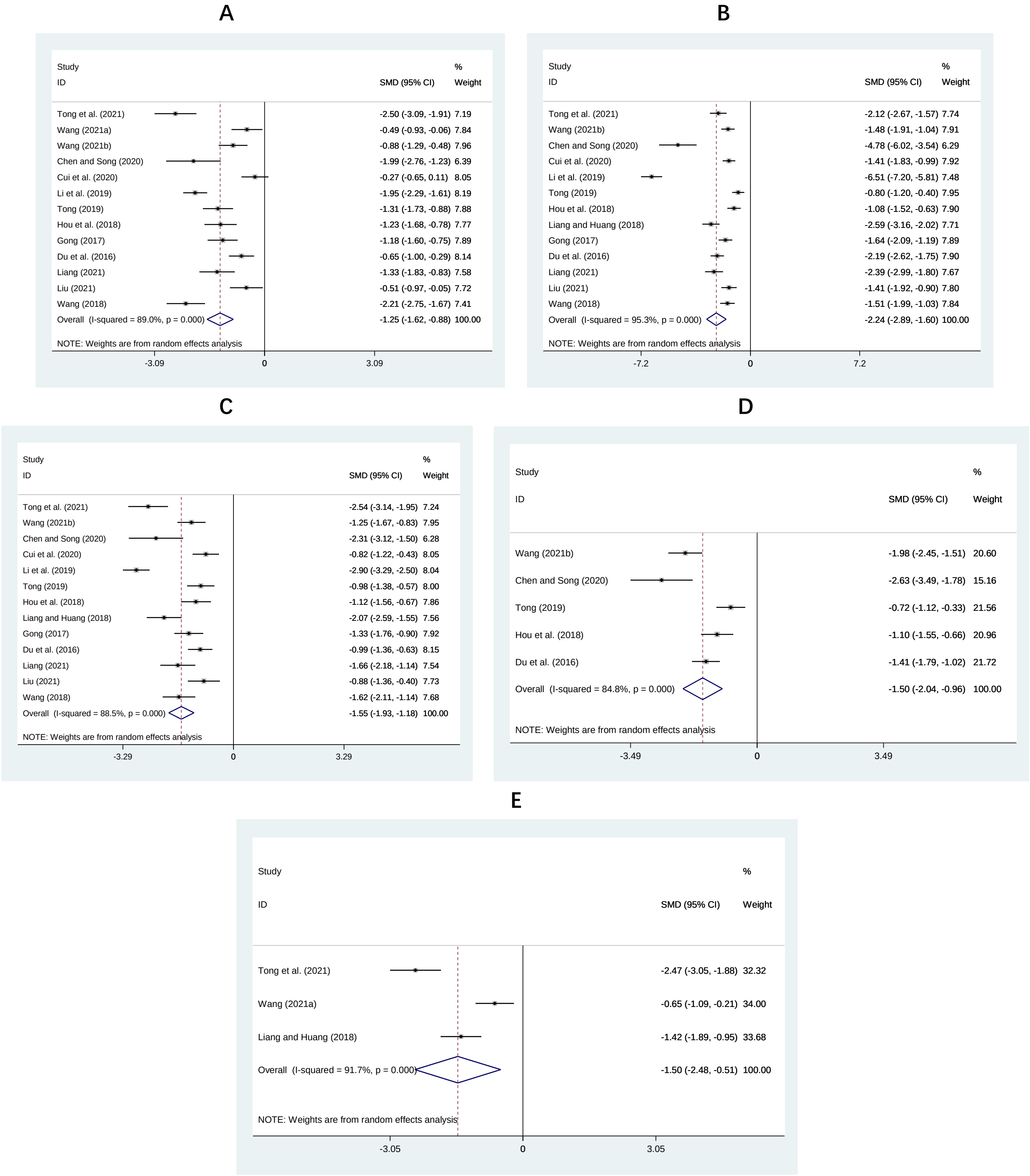

Figure 6.2. Results of meta-analysis for outcomes of clinical symptom indexes. A, Time to disappearance of fever; B, Time to disappearance of cough; C, Time to disappearance of lung rales; D, Time to relief of asthma; E, Time for return to normal of chest X-ray.

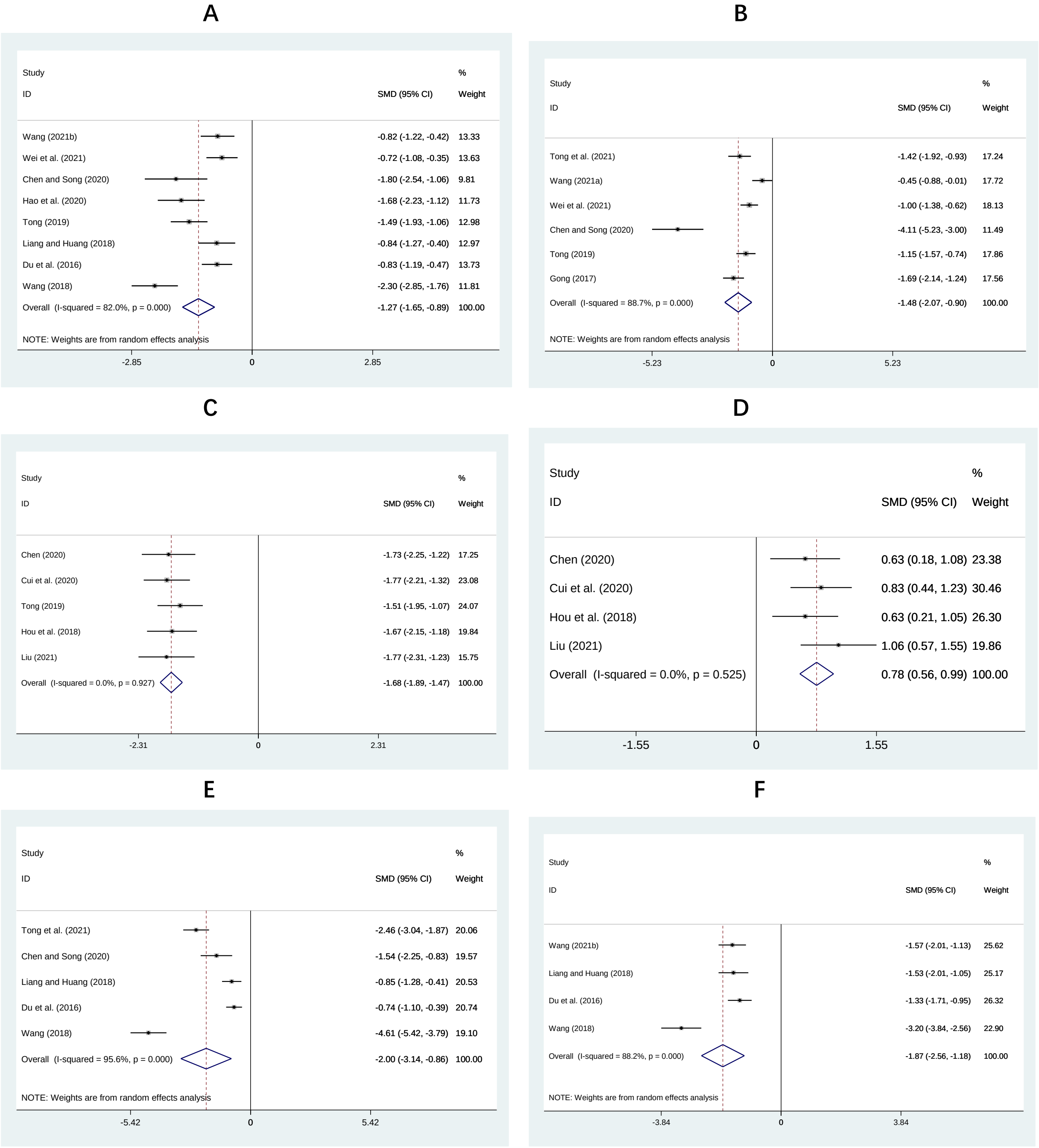

Figure 6.3. Results of meta-analysis for outcomes of inflammatory indicators. A, Tumor necrosis factor alpha (TNF-α); B, C-reaction protein (CRP); C, Interleukin-2 (IL-2); D, Interleukin-4 (IL-4); E, Interleukin-6 (IL-6); F, Interleukin-8 (IL-8).

Appendix S7 Subgroup analysis

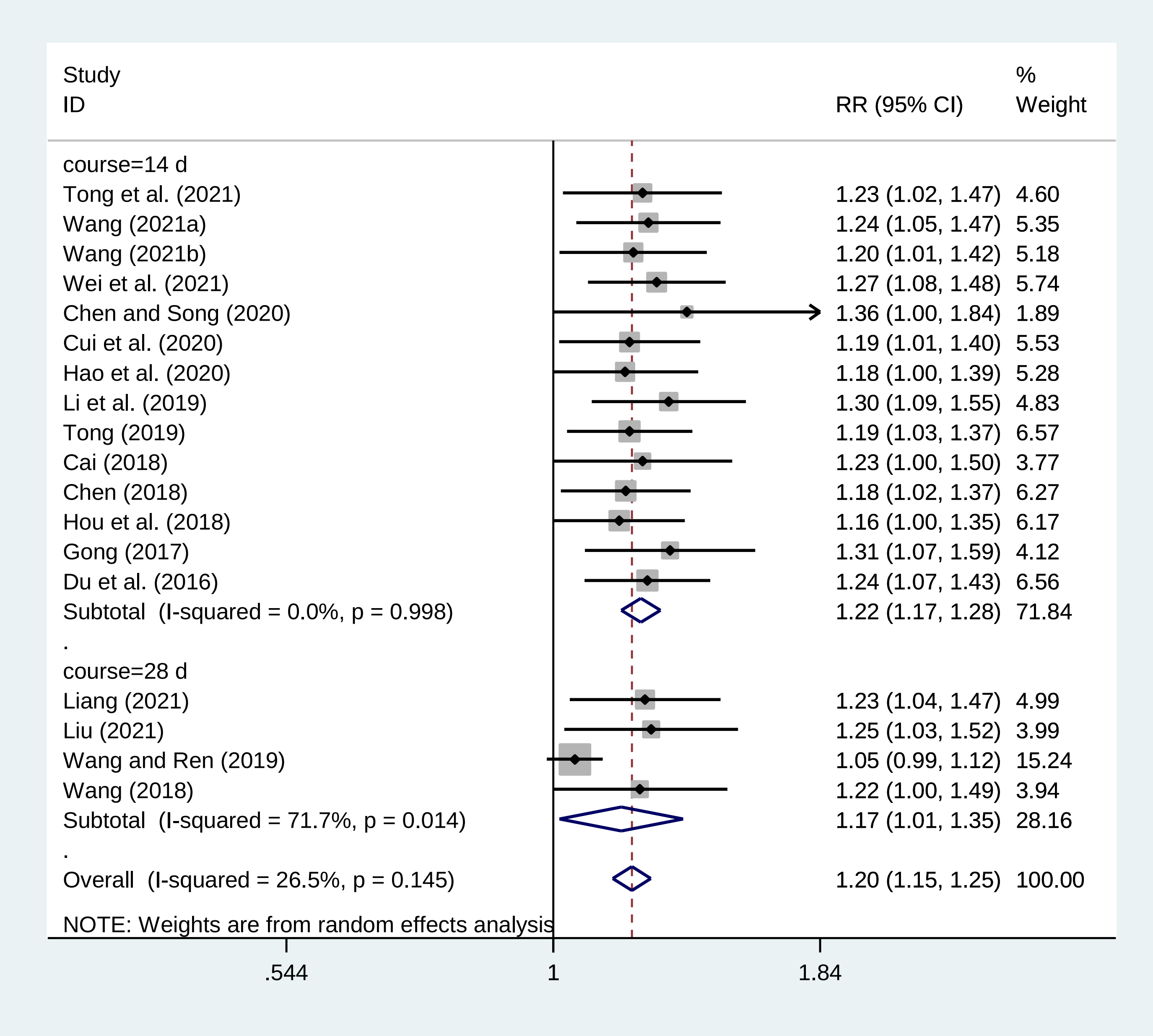

Figure S7. Meta-analysis forest plot comparing TER of the intervention group with the control group for subgroup analysis of different treatment duration.

Appendix S8 Sensitivity analysis

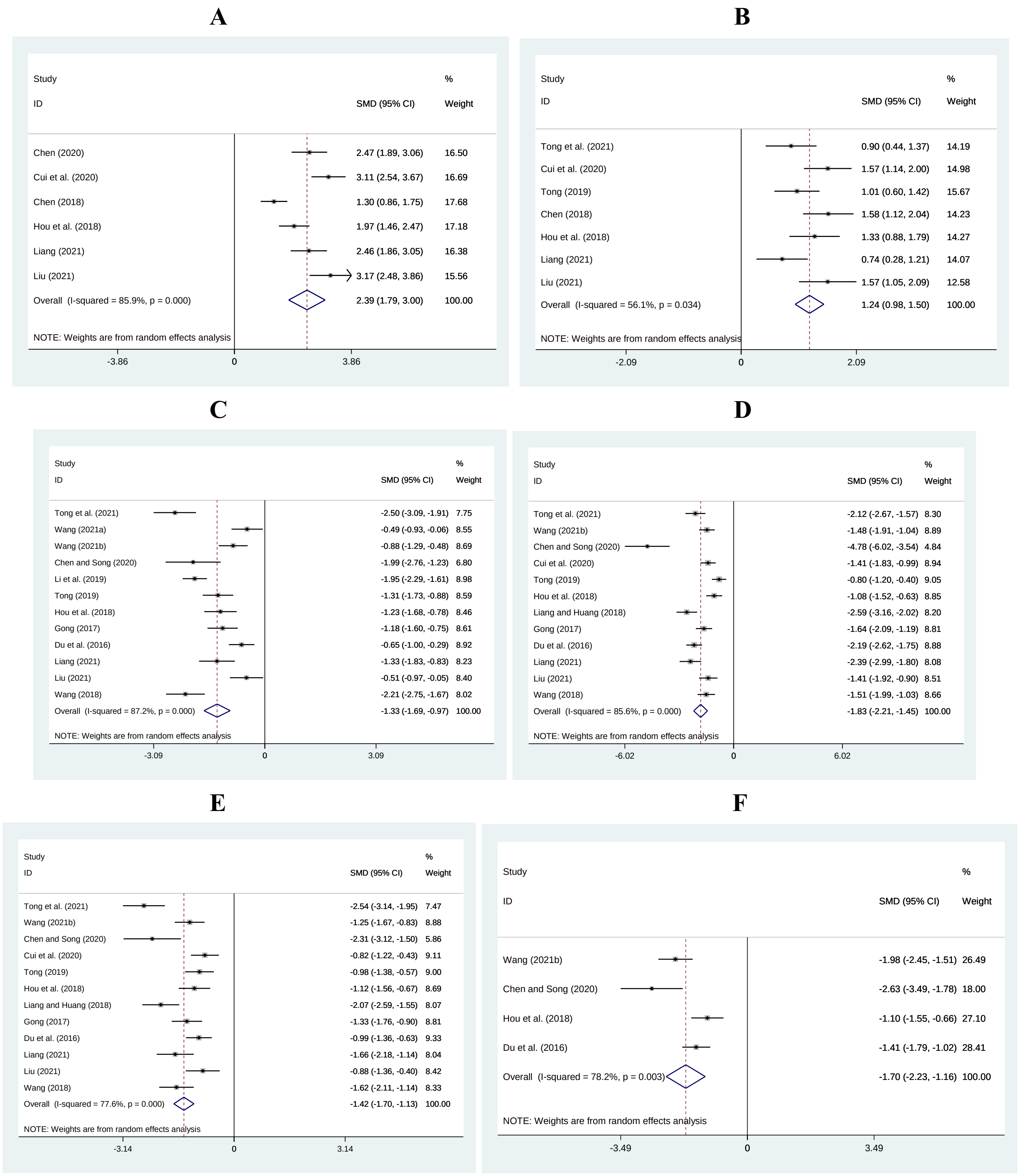

Figure S8.1. Sensitivity analysis of the cumulative meta-analysis results regarding outcomes of pulmonary function indexes and clinical symptom indexes. A, Ratio of Forced expiratory volume in one second / Forced vital capacity (FEV1 / FVC) after removing the study done by Tong (2019); B, Peak expiratory flow (PEF) after removing the study done by Chen (2020); C, Time to disappearance of fever after removing the study done by Cui et al. (2020); D, Time to disappearance of cough after removing the study done by Li et al. (2019); E, Time to disappearance of lung rales after removing the study done by Li et al. (2019); F, Time to relief of asthma after removing the study done by Tong (2019).

A

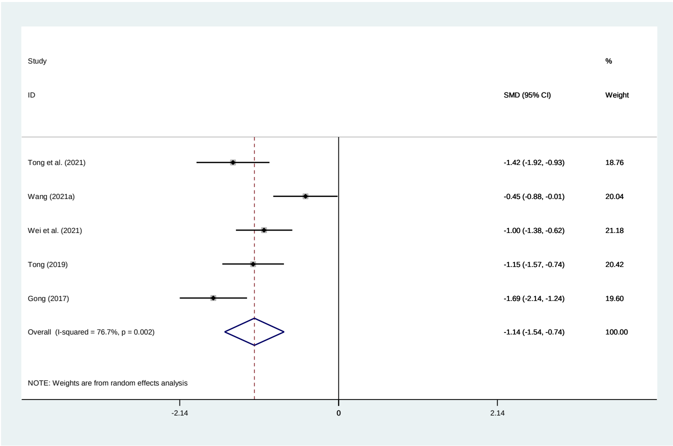

B

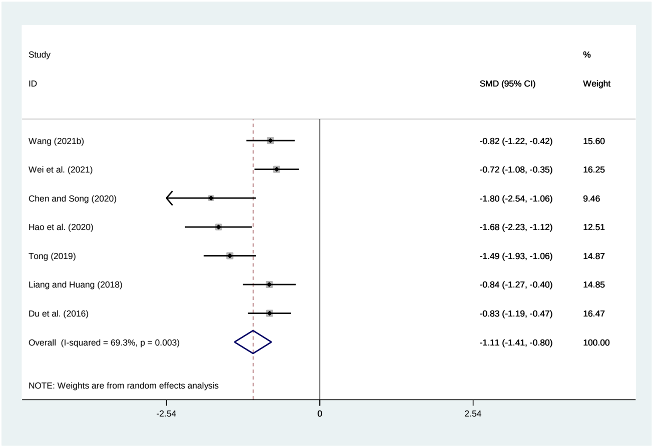

C

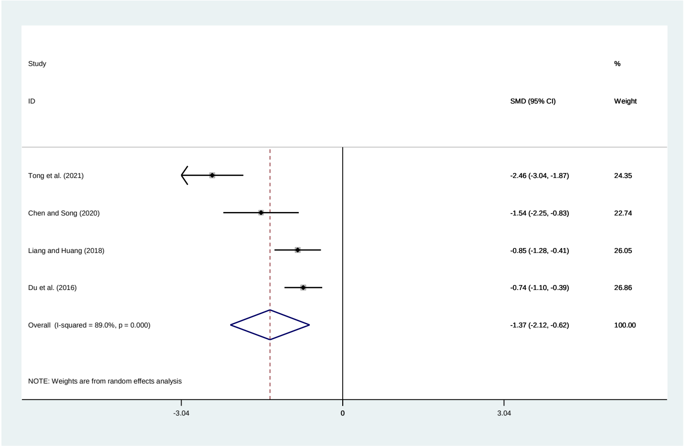

D

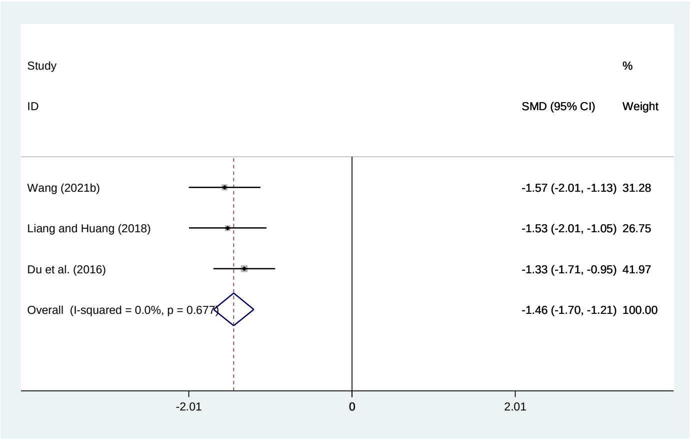

Figure S8.2. Sensitivity analysis of the cumulative meta-analysis results regarding outcomes of inflammatory indicators. A, C-reaction protein (CRP) after removing the study done by Chen and Song (2020); B, Tumor necrosis factor alpha (TNF-α) after removing the study done by Wang (2018); C, Interleukin-6 (IL-6) after removing the study done by Wang (2018); D, Interleukin-8 (IL-8) after removing the study done by Wang (2018).

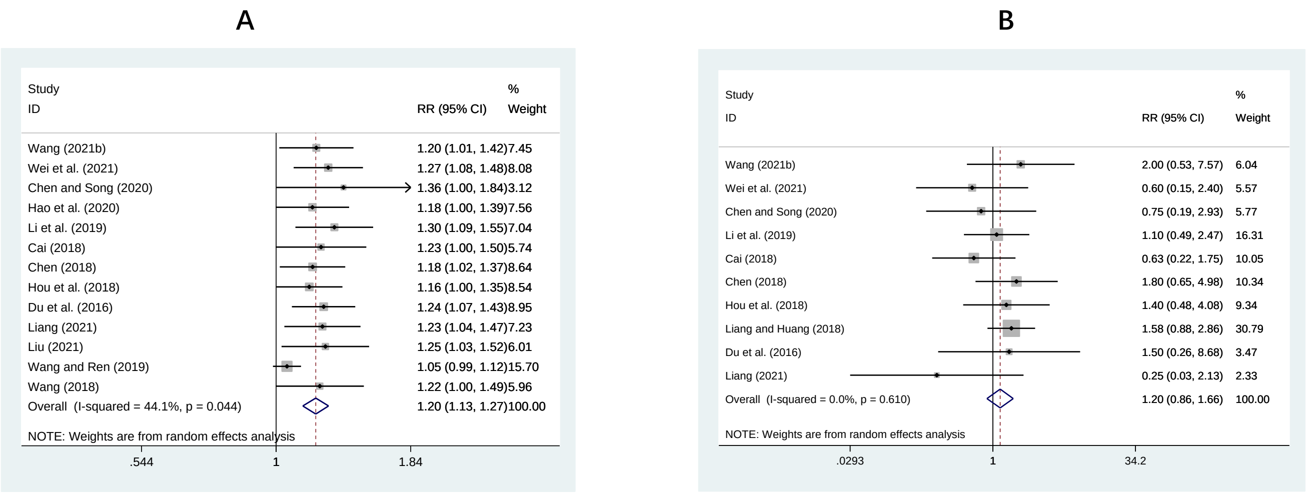

Figure S8.3. Sensitivity analysis for primary outcomes based on risk of bias (excluding studies at high risk of bias). A, Total effectiveness rate (TER); B, Incidence of total adverse events (TAEs). Studies done by Tong et al. (2021), Wang (2021a), Chen (2020), Cui et al. (2020), Tong (2019) and Gong (2017) were at high risk of bias.

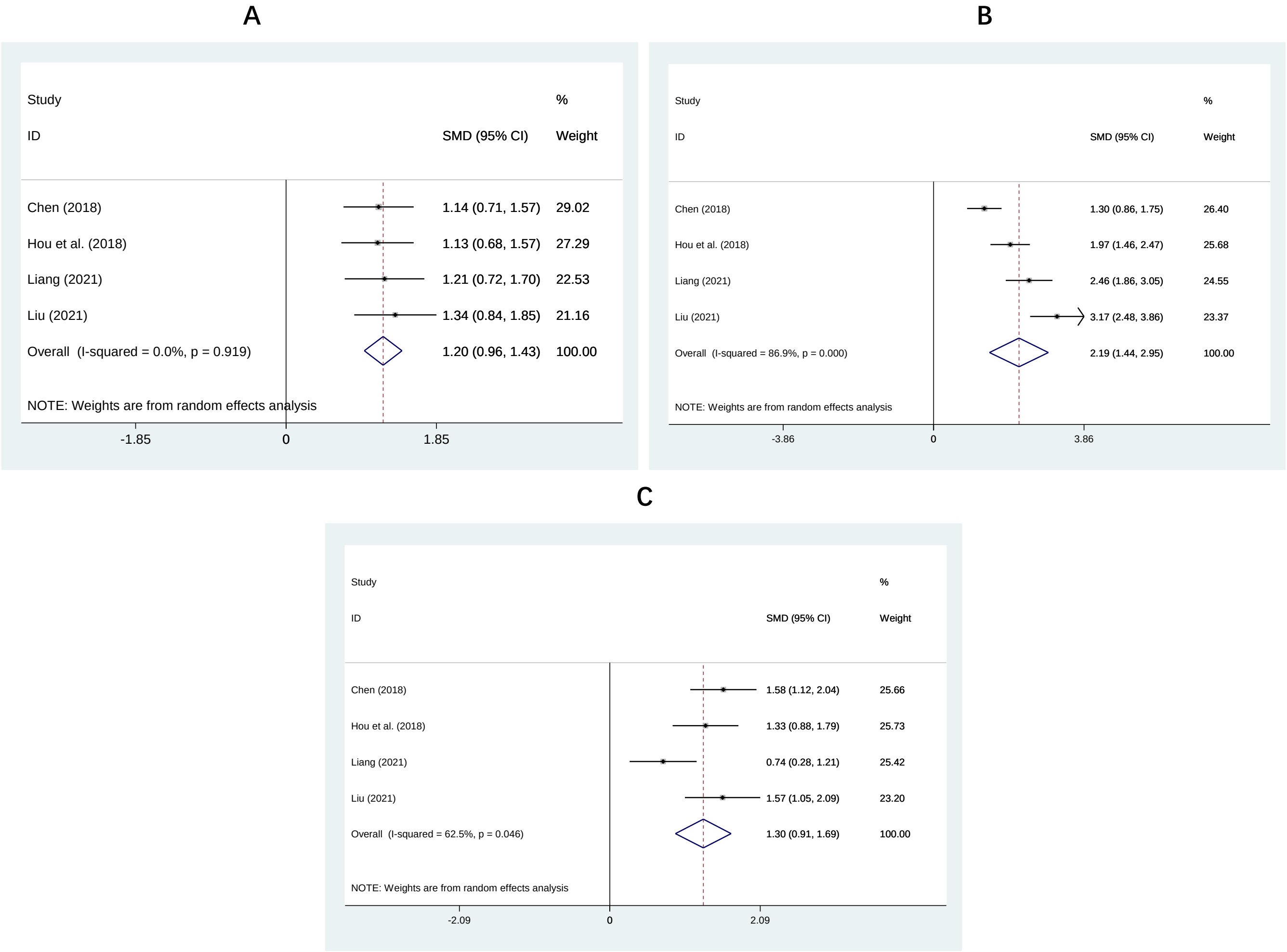

Figure S8.4. Sensitivity analysis for outcomes of pulmonary function indexes based on risk of bias (excluding studies at high risk of bias). A, Forced expiratory volume in one second (FEV1); B, the ratio of Forced expiratory volume in one second / Forced vital capacity (FEV1 / FVC); C, Peak expiratory flow (PEF). Studies done by Tong et al. (2021), Wang (2021a), Chen (2020), Cui et al. (2020), Tong (2019) and Gong (2017) were at high risk of bias.

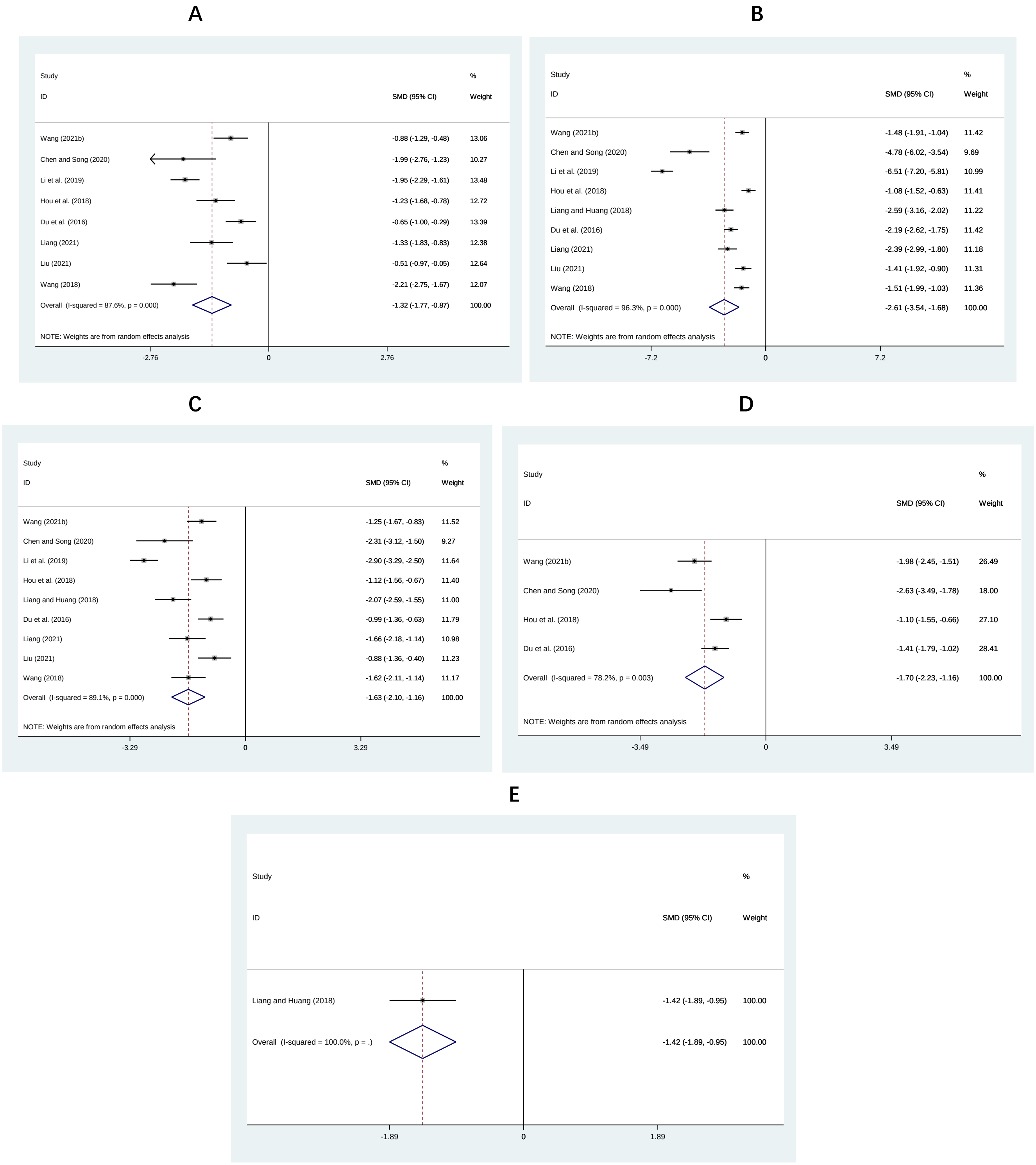

Figure S8.5. Sensitivity analysis for outcomes of clinical symptom indexes based on risk of bias (excluding studies at high risk of bias). A, Time to disappearance of fever; B, Time to disappearance of cough; C, Time to disappearance of lung rales; D, Time to relief of asthma; E, Time for return to normal of chest X-ray. Studies done by Tong et al. (2021), Wang (2021a), Chen (2020), Cui et al. (2020), Tong (2019) and Gong (2017) were at high risk of bias.

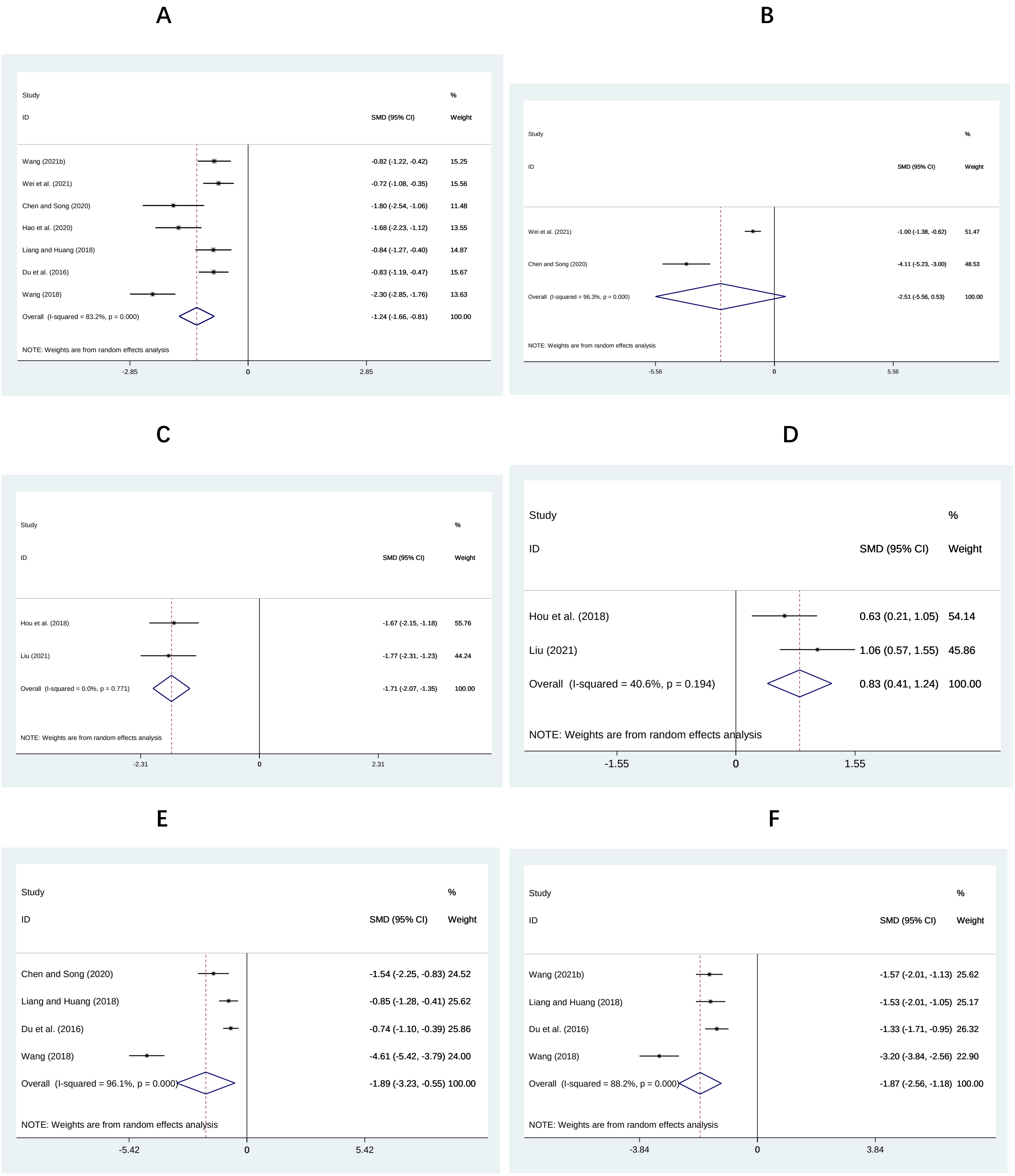

Figure S8.6. Sensitivity analysis for outcomes of inflammatory indicators based on risk of bias (excluding studies at high risk of bias). A, Tumor necrosis factor alpha (TNF-α); B, C-reaction protein (CRP); C, Interleukin-2 (IL-2); D, Interleukin-4 (IL-4); E, Interleukin-6 (IL-6); F, Interleukin-8 (IL-8). Studies done by Tong et al. (2021), Wang (2021a), Chen (2020), Cui et al. (2020), Tong (2019) and Gong (2017) were at high risk of bias.

Appendix S9 Publication bias

A B

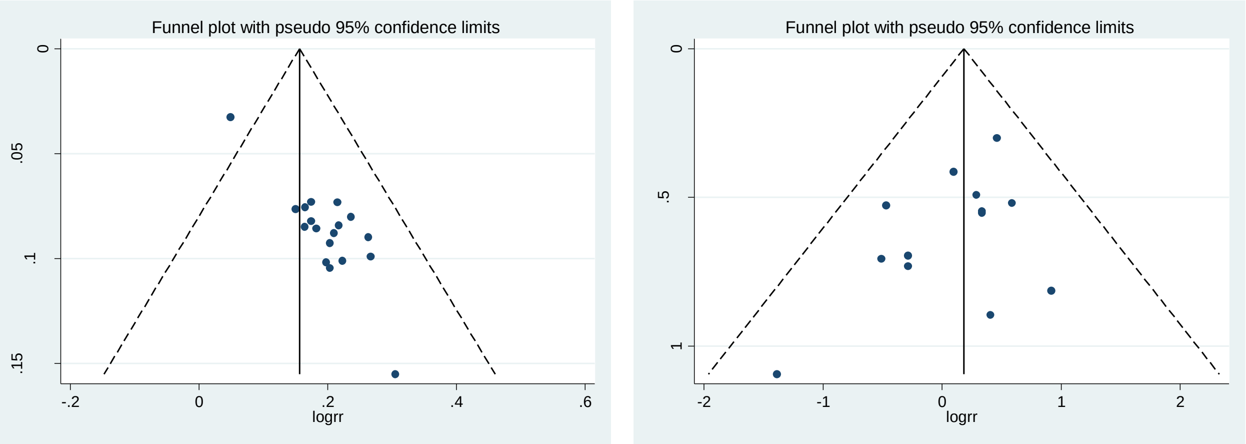

Figure S9.1. Funnel plot. A, Total effectiveness rate (TER); B, the incidence of Total adverse events (TAEs).

A B

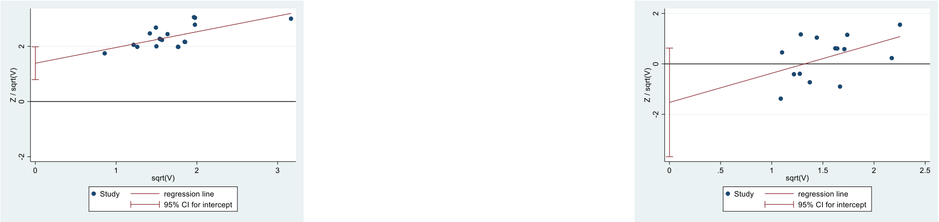

Figure S9.2. Harbord-weighted linear regression. A, Total effectiveness rate (TER, t=4.94, $P < 0.001$); B, the incidence of Total adverse events (TAEs, t=-1.55, $P = 0.148$).

Appendix S10 Summary of adverse events

Table S9. Different adverse events in the included RCTs

RCTs	Nausea and vomiting [n (%)]		Abdominal distension, abdominal pain, diarrhea [n (%)]		Headache [n (%)]		Rash [n (%)]		Angioneurotic edema [n (%)]		Oral fungal infection [n (%)]		Arrhythmia [n (%)]		Observation patients [n]	
	I	C	I	C	I	C	I	C	I	C	I	C	I	C	I	C
Tong et al., 2021	5 (12.5)	3 (7.5)	3 (7.5)	3 (7.5)	\	\	\	\	\	\	\	\	\	\	40	40
Wang, 2021a	1 (2.4)	1 (2.4)	2 (4.8)	2 (4.8)	\	\	0 (0.0)	1 (2.4)	\	\	\	\	\	\	42	42
Wang, 2021b	3 (5.8)	2 (3.8)	\	\	\	\	2 (3.8)	1 (1.9)	\	\	1 (1.9)	0 (0.0)	\	\	52	52
Wei et al., 2021	2 (3.3)	2 (3.3)	1 (1.7)	2 (3.3)	\	\	0 (0.0)	1 (1.7)	\	\	\	\	\	\	60	60
Chen and Song, 2020	1 (5.0)	2 (10.0)	1 (5.0)	1 (5.0)	1 (5.0)	1 (5.0)	\	\	\	\	\	\	\	\	20	20
Cui et al., 2020	5 (9.3)	3 (5.6)	1 (1.9)	1 (1.9)	1 (1.9)	0 (0.0)	0 (0.0)	1 (1.9)	\	\	\	\	\	\	54	54
Li et al., 2019	5 (5.0)	4 (4.0)	3 (3.0)	2 (2.0)	2 (2.0)	2 (2.0)	2 (2.0)	2 (2.0)	\	\	\	\	\	\	100	100
Tong, 2019	1 (1.9)	1 (1.9)	1 (1.9)	0 (0.0)	2 (3.8)	1 (1.9)	\	\	\	\	\	\	1 (1.9)	0 (0.0)	52	52
Cai, 2018	2 (4.8)	3 (7.1)	1 (2.4)	2 (4.8)	\	\	2 (4.8)	3 (7.1)	\	\	\	\	\	\	42	42
Chen, 2018	7 (14.6)	4 (8.3)	2 (4.2)	1 (2.1)	\	\	\	\	\	\	\	\	\	\	48	48
Hou et al., 2018	4 (8.9)	3 (6.7)	2 (4.4)	2 (4.4)	1 (2.2)	0 (0.0)	\	\	\	\	\	\	\	\	45	45
Liang and Huang, 2018	5 (11.4)	3 (6.8)	5 (11.4)	4 (9.1)	6 (13.6)	3 (6.8)	\	\	3 (6.8)	2 (4.5)	\	\	\	\	44	44
Du et al., 2016	3 (4.6)	2 (3.1)	\	\	\	\	\	\	\	\	\	\	\	\	65	65
Liang, 2021	1 (2.6)	1 (2.6)	0 (0.0)	1 (2.6)	0 (0.0)	2 (5.3)	\	\	\	\	\	\	\	\	38	38
**Total**	45 (6.4)	34 (4.8)	22 (3.1)	21 (3)	13 (1.9)	9 (1.3)	6 (0.9)	9 (1.3)	3 (0.4)	2 (0.3)	1 (0.1)	0 (0.0)	1 (0.1)	0 (0.0)	702	702

Note: I, Intervention group; C, Control group; “\” denotes “not included”.

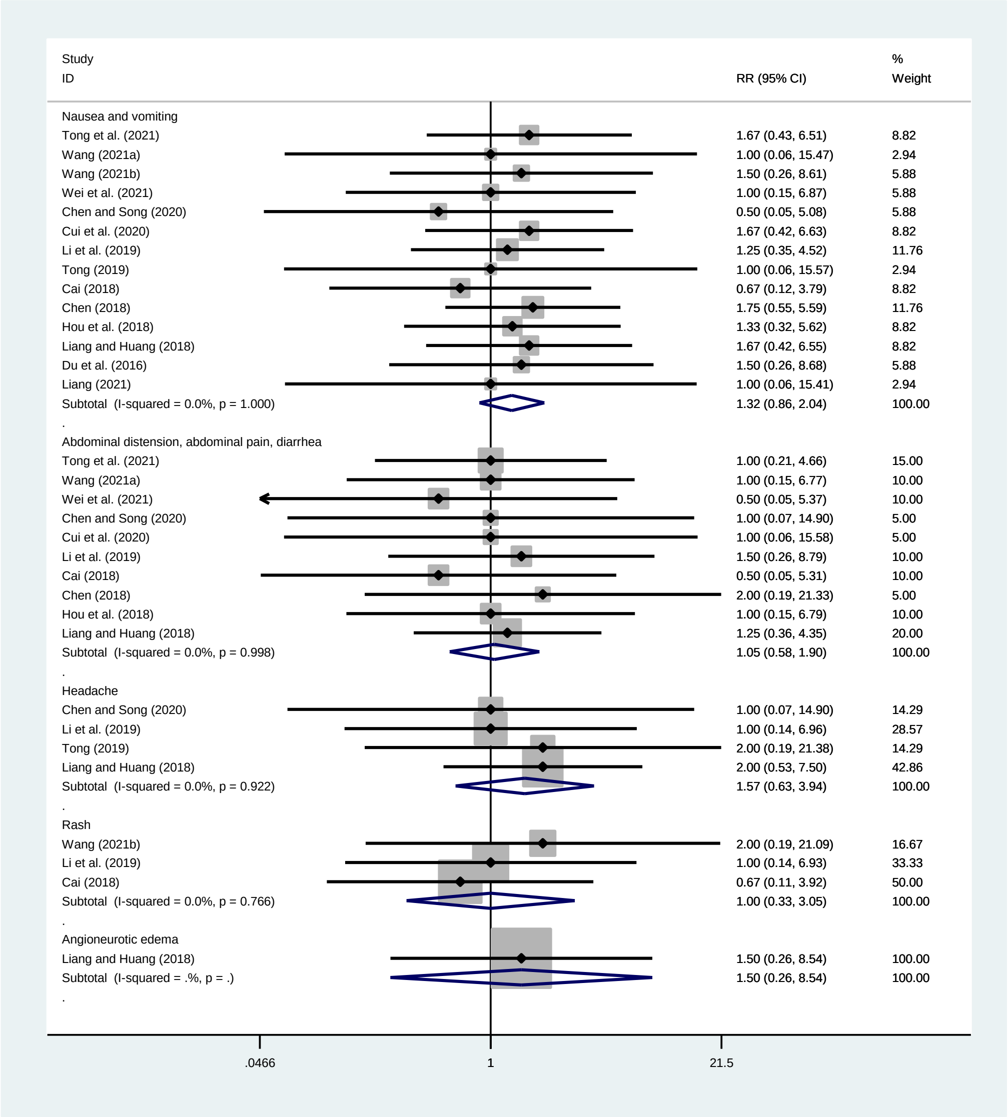

Figure S10. Results of meta-analysis for incidence of different adverse events.

Appendix S11 PRISMA 2020 Checklist

Section and Topic	Item #	Checklist item	Location where item is reported
**TITLE**			
Title	1	Identify the report as a systematic review.	1
**ABSTRACT**			
Abstract	2	See the PRISMA 2020 for Abstracts checklist.	2-3
**INTRODUCTION**			
Rationale	3	Describe the rationale for the review in the context of existing knowledge.	3-4
Objectives	4	Provide an explicit statement of the objective(s) or question(s) the review addresses.	4
**METHODS**			
Eligibility criteria	5	Specify the inclusion and exclusion criteria for the review and how studies were grouped for the syntheses.	4-6
Information sources	6	Specify all databases, registers, websites, organisations, reference lists and other sources searched or consulted to identify studies. Specify the date when each source was last searched or consulted.	6-7
Search strategy	7	Present the full search strategies for all databases, registers and websites, including any filters and limits used.	6-7, Appendix S1
Selection process	8	Specify the methods used to decide whether a study met the inclusion criteria of the review, including how many reviewers screened each record and each report retrieved, whether they worked independently, and if applicable, details of automation tools used in the process.	7
Data collection process	9	Specify the methods used to collect data from reports, including how many reviewers collected data from each report, whether they worked independently, any processes for obtaining or confirming data from study investigators, and if applicable, details of automation tools used in the process.	7
Data items	10a	List and define all outcomes for which data were sought. Specify whether all results that were compatible with each outcome domain in each study were sought (e.g. for all measures, time points, analyses), and if not, the methods used to decide which results to collect.	7

Section and Topic	Item #	Checklist item	Location where item is reported
	10b	List and define all other variables for which data were sought (e.g. participant and intervention characteristics, funding sources). Describe any assumptions made about any missing or unclear information.	7
Study risk of bias assessment	11	Specify the methods used to assess risk of bias in the included studies, including details of the tool(s) used, how many reviewers assessed each study and whether they worked independently, and if applicable, details of automation tools used in the process.	7
Effect measures	12	Specify for each outcome the effect measure(s) (e.g. risk ratio, mean difference) used in the synthesis or presentation of results.	7-8
Synthesis methods	13a	Describe the processes used to decide which studies were eligible for each synthesis (e.g. tabulating the study intervention characteristics and comparing against the planned groups for each synthesis (item #5)).	7-8
	13b	Describe any methods required to prepare the data for presentation or synthesis, such as handling of missing summary statistics, or data conversions.	NA
	13c	Describe any methods used to tabulate or visually display results of individual studies and syntheses.	7-8
	13d	Describe any methods used to synthesize results and provide a rationale for the choice(s). If meta-analysis was performed, describe the model(s), method(s) to identify the presence and extent of statistical heterogeneity, and software package(s) used.	7-8
	13e	Describe any methods used to explore possible causes of heterogeneity among study results (e.g. subgroup analysis, meta-regression).	7-8
	13f	Describe any sensitivity analyses conducted to assess robustness of the synthesized results.	7-8
Reporting bias assessment	14	Describe any methods used to assess risk of bias due to missing results in a synthesis (arising from reporting biases).	8
Certainty assessment	15	Describe any methods used to assess certainty (or confidence) in the body of evidence for an outcome.	8
**RESULTS**			
Study selection	16a	Describe the results of the search and selection process, from the number of records identified in the search to the number of studies	8-9, Figure 1,

Section and Topic	Item #	Checklist item	Location where item is reported
		included in the review, ideally using a flow diagram.	Appendix S2
	16b	Cite studies that might appear to meet the inclusion criteria, but which were excluded, and explain why they were excluded.	Figure 1
Study characteristics	17	Cite each included study and present its characteristics.	8-9, Table 1, Appendix S3,4
Risk of bias in studies	18	Present assessments of risk of bias for each included study.	9, Figure 2, Appendix S5
Results of individual studies	19	For all outcomes, present, for each study: (a) summary statistics for each group (where appropriate) and (b) an effect estimate and its precision (e.g. confidence/credible interval), ideally using structured tables or plots.	9, Table 1
Results of syntheses	20a	For each synthesis, briefly summarise the characteristics and risk of bias among contributing studies.	9, Table 1
	20b	Present results of all statistical syntheses conducted. If meta-analysis was done, present for each the summary estimate and its precision (e.g. confidence/credible interval) and measures of statistical heterogeneity. If comparing groups, describe the direction of the effect.	10-12, Figure 3-4, Table 2-3, Appendix S6,10
	20c	Present results of all investigations of possible causes of heterogeneity among study results.	12-13
	20d	Present results of all sensitivity analyses conducted to assess the robustness of the synthesized results.	12-13, Appendix S7-8
Reporting biases	21	Present assessments of risk of bias due to missing results (arising from reporting biases) for each synthesis assessed.	13, Appendix S9
Certainty of evidence	22	Present assessments of certainty (or confidence) in the body of evidence for each outcome assessed.	13, Table 4
**DISCUSSION**			
Discussion	23a	Provide a general interpretation of the results in the context of other evidence.	14

Section and Topic	Item #	Checklist item	Location where item is reported
	23b	Discuss any limitations of the evidence included in the review.	15-16
	23c	Discuss any limitations of the review processes used.	15-16
	23d	Discuss implications of the results for practice, policy, and future research.	17
**OTHER INFORMATION**			
Registration and protocol	24a	Provide registration information for the review, including register name and registration number, or state that the review was not registered.	4, the registered protocol (CRD42023452139)
	24b	Indicate where the review protocol can be accessed, or state that a protocol was not prepared.	4
	24c	Describe and explain any amendments to information provided at registration or in the protocol.	NA
Support	25	Describe sources of financial or non-financial support for the review, and the role of the funders or sponsors in the review.	19
Competing interests	26	Declare any competing interests of review authors.	19
Availability of data, code and other materials	27	Report which of the following are publicly available and where they can be found: template data collection forms; data extracted from included studies; data used for all analyses; analytic code; any other materials used in the review.	18

From: Page MJ, McKenzie JE, Bossuyt PM, Boutron I, Hoffmann TC, Mulrow CD, et al. The PRISMA 2020 statement: an updated guideline for reporting systematic reviews. BMJ 2021;372:n71. doi: 10.1136/bmj.n71

For more information, visit: http://www.prisma-statement.org/
